# Pediatric patient engagement in clinical care, research and intervention development: a scoping review

**DOI:** 10.1186/s41687-023-00566-y

**Published:** 2023-03-29

**Authors:** Lorynn Teela, Lieke E. Verhagen, Hedy A. van Oers, Esmée E. W. Kramer, Joost G. Daams, Mariken P. Gruppen, Maria J. Santana, Martha A. Grootenhuis, Lotte Haverman

**Affiliations:** 1 Amsterdam UMC location University of Amsterdam, Emma Children’s Hospital, Child and Adolescent Psychiatry & Psychosocial Care, Meibergdreef 9, Amsterdam, The Netherlands; 2 Amsterdam Public Health, Mental health and Digital health, Amsterdam, The Netherlands; 3 Amsterdam Reproduction and Development, Child development, Amsterdam, The Netherlands; 4 Amsterdam UMC location University of Amsterdam, Research Support, Medical Library, Meibergdreef 9, Amsterdam, The Netherlands; 5 Amsterdam UMC location University of Amsterdam, Emma Children’s Hospital, Department of General Pediatrics, Meibergdreef 9, Amsterdam, The Netherlands; 6 Department of Community Health Sciences, Cumming School of Medicine, University of Calgary, Calgary, AB Canada; 7 Department of Pediatrics, Cumming School of Medicine, University of Calgary, Calgary, AB Canada; 8 Princess Maxima Center for Pediatric Oncology, Utrecht, The Netherlands

**Keywords:** Patient participation, Patient involvement, Pediatrics, Chronic diseases

## Abstract

**Background:**

In the last decades, pediatric patient engagement has received growing attention and its importance is increasingly acknowledged. Pediatric patient engagement in health care can be defined as the involvement of children and adolescents in the decision-making of daily clinical care, research and intervention development. Although more attention is paid to pediatric patient engagement, a comprehensive overview of the activities that have been done regarding pediatric patient engagement and the changes over time is lacking. Therefore, the aim of this study is to provide an overview of the literature about pediatric patient engagement.

**Methods:**

The methodological framework of Arksey & O’Malley was used to conduct this scoping review. The bibliographic databases Medline, Embase, and PsycINFO were searched for eligible articles. All retrieved articles were screened by at least two researchers in two steps. Articles were included if they focused on pediatric patient engagement, were carried out in the context of clinical care in pediatrics, and were published as full text original article in English or Dutch. Data (year of publication, country in which the study was conducted, disease group of the participants, setting of pediatric patient engagement, used methods, and age of participants) were extracted, synthesized, and tabulated.

**Results:**

A total of 288 articles out of the 10,714 initial hits met the inclusion criteria. Over the years, there has been an increase in the number of studies that engage pediatric patients. Pediatric patients, especially patients with multiple conditions or oncology patients, were most involved in studies in the United States, United Kingdom, and Canada. Pediatric patients were most often asked to express their views on questions from daily clinical care and the individual interview was the most used method. In general, the extent to which pediatric patients are engaged in health care increases with age.

**Discussion:**

This scoping review shows that there is an increasing interest in pediatric patient engagement. However, lack of uniformity about the definition of pediatric patient engagement and clear information for clinicians hinders engagement. This overview can inform clinicians and researchers about the different ways in which pediatric patient engagement can be shaped and can guide them to engage pediatric patients meaningfully in their projects.

**Supplementary Information:**

The online version contains supplementary material available at 10.1186/s41687-023-00566-y.

## Introduction

In 1989, over 190 countries, including the Netherlands, signed the United Nations Convention on the Rights of the Child (UNCRC) [1]. The UNCRC describes the human rights for every child, such as self-determination, freedom of thoughts and religion, and the right to have a say in matters that affect them. It is with this convention that the engagement of children in health care, research and intervention development became more important [2, 3]. From that moment on, clinicians, researchers and policymakers more often tried to carry out their health care projects and decision-making together *with* pediatric patients rather than *about* or *for* pediatric patients [3].

Involving children in decision-making about daily clinical care, research and intervention development is referred to as ‘pediatric patient engagement’ [4, 5]. The extent to which children influence the decision-making processes can vary from consultation (e.g., patients are asked for their opinion, but have limited influences on decision-making) to active partnership (e.g., patients cooperate as equal partners with other stakeholders and share responsibility) [6, 7]. Notwithstanding the extent of involvement, pediatric patient engagement has important value for health care. Previous research shows that pediatric patient engagement increases children’s self-confidence and sense of control, which results in better treatment outcomes [8]. Moreover, pediatric patient engagement leads to higher inclusion rates in research and improves the translation from research to clinical practice [9].

Although the importance of pediatric patient engagement is acknowledged, pediatric patients are not always involved in the decision-making process in health care [8, 10]. Clinicians, researchers, and policymakers are, for example, reserved in involving pediatric patients in health care as they doubt the capacity of children required for participating, and they lack experience in engaging children [8, 11, 12]. In addition, pediatric patient engagement is complicated by the tendency of adults to protect children from making difficult decisions [8, 11]. Professionals therefore need more support to involve pediatric patients meaningfully and usefully [13].

In the last years, a few systematic reviews on pediatric patient engagement in clinical care have been conducted [2, 3, 14]. These systematic reviews are relatively outdated (over 10 years old), given the fact that pediatric participation is a developing practice. The focus of the conducted systematic reviews were only on engagement in the decision-making process in the consultation room and the challenges involved [2, 14]. Also, in one paper, the included articles are only summarized and interpreted by one author [2], as opposed to systematically collating, summarizing, and reporting the results. A recent scoping review describes the involvement of adolescents and young adults (12–25 years) with a chronic condition in health and social care [3]. This review, only including 23 studies, provides a synopsis of the used definitions of patient engagement, goals, methods, and impact of the involvement of youth in research and implementation projects. However, a comprehensive overview of the activities that have been done in the past regarding pediatric patient engagement, also including primary school-aged children (4–18 years) in health care is lacking, as well as insights into how patient engagement takes place in clinical care, research, and intervention development. In addition, we want to know how pediatric patient engagement has developed in recent years to learn more about the different ways pediatric patients can be involved in health care. Therefore, the aim of this study is to provide an overview of the literature about pediatric patient engagement in clinical care, research, and intervention development.

## Methods

Due to the broad nature of the study aim, a scoping review was conducted. Scoping reviews can be used to provide an overview and map the available evidence around a certain topic [15, 16]. The methodological framework of Arksey & O’Malley [16] was used to guide this scoping review. This framework consisted of the following 5 stages:

### Stage 1: identifying the research question

The research question of this scoping review was: *What is known from the literature about pediatric patient engagement in clinical care, research and intervention development?* A comprehensive approach was chosen to examine the extent and nature of pediatric patient engagement in the broad field of pediatrics. Key parameters were **patient engagement** (defined as: actively involving children in the clinical care, medical research, and intervention development. This means that children were asked for their opinion on certain topics or that they played a role in the decision-making process), **children and adolescents** (defined as people aged 4–18 years), and **pediatrics** (defined as the medical care of children and adolescents in a hospital/clinical setting and the associated science).

### Stage 2: identifying relevant studies

A comprehensive search strategy was developed and carried out in collaboration with a medical research librarian (JGD). To obtain a clear description of the construct, both published and unpublished literature about engagement of children and adolescents was collected and reviewed by at least two research-psychologists (FW, MV, LH). Subsequently, a visualization of similarities (VOS) analysis [17] was carried out with the software tool VOSviewer® to remove irrelevant terms from the search strategy by NOTing [18]. Medline, Embase and PsycINFO were searched for eligible articles from inception (May 2017). The construct of the search strategy can be summarized as follows: ([hospitalized patient] AND [patient participation]) NOT [irrelevant terms identified by VOS analysis]. See Additional file 1 for full search details.

In February 2021, an update of the literature search was done. The same search strategy was applied. The bibliographic databases were searched for eligible articles in the period January 2017 until February 2021. For practical reasons, duplicate articles from the period January 2017 to May 2017 were removed in the last step of the study selection.

### Stage 3: study selection

Title and abstract of the articles retrieved were assessed by at least two members of the research team (LT, LEV, EEWK, FW, MV, LH) using the software tool Rayyan [19]. To reduce individual bias during the screening process and to refine inclusion and exclusion criteria, consultation took place between the members of the research team after screening the first 300 articles. The full text of potentially relevant articles was obtained and assessed by at least two members of the team (LT, LEV, EEWK). If necessary, a third member (LH) made the decision regarding inclusion of an article. An article was included if the study described all following inclusion criteria:

- Focused on engagement of children and adolescents (4–18 years). Studies that included pediatric patients in a broader age range or studies that included both pediatric patients and young adults were also included.
- Participants were asked for their opinion regarding clinical care, research, policy and/or intervention development.
- Carried out in the context of clinical care/pediatrics.
- Published as a full text original article (i.e. not an abstract, review, commentary, dissertation or study protocol).
- Published in English or Dutch.

Studies that reported only on the engagement of representatives of pediatric patients (i.e., caregivers, family members) or studies that did not clearly distinguish pediatric patients as a subgroup were excluded. In addition, studies that were conducted in the field of dentistry or psychiatry or studies that described the engagement of pediatric patients in a school or home setting were excluded. Also, studies that explored the experiences of children living with a medical condition in general (e.g., experiences of children living with HIV) were excluded, unless the studies reported on the life-experiences of these children with the aim to improve a medical treatment or to develop an intervention/tool. Furthermore, studies describing the involvement of pediatric patients in developing measurements using cognitive interviews for checking the understanding of questions or icons were excluded. The research team does not consider using cognitive interviews for this purpose to be part of pediatric patient engagement. The opinion of children and adolescents is thus not being asked in these cognitive interviews. Finally, studies that only described the importance of pediatric patient engagement, but did not discuss the application of pediatric patient engagement, were also excluded.

### Stage 4: charting the data

A data extraction form was developed by the team, and data were extracted from the included articles by one members of the team (LT, LEV, or EEWK). A second member of the team (LT, LEV, or EEWK) cross-checked a selection of the extracted data. The following data were extracted from the articles: year of publication, country in which the study was conducted, disease group of the participants, number of participants, setting of pediatric patient engagement (health care, research, or development of interventions or tools), method used for patient engagement, and age of participants.

### Stage 5: collating, summarizing, and reporting the results

Extracted data were analyzed quantitatively with the use of the Statistical Package for Social Sciences (SPSS) version 28. This quantitative data provided an overview of the nature and extent of pediatric patient engagement. To learn more about the goals of pediatric patient engagement, the data were screened by the research team and examples were cited.

## Results

### Search and selection results

**Fig. 1:**
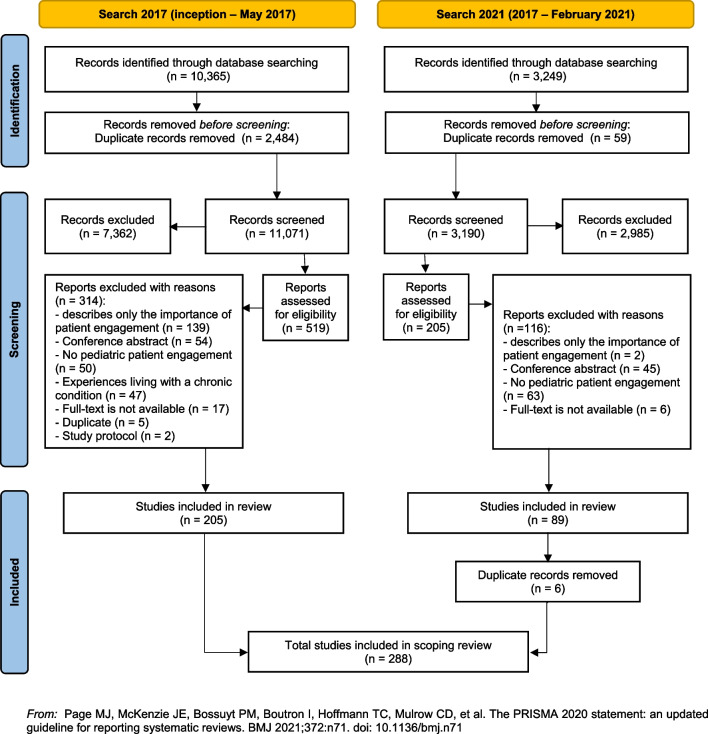
Flow chart of the identification and selection process of studies [20]

The study selection process is presented in the PRISMA flow diagram of Fig. 1. The literature search yielded 10,365 (2017) and 3249 (2021) articles. After removing duplicates, title and abstracts of 11,071 (2017) and 3190 (2021) articles were assessed. Of these, 519 (2017) and 205 articles (2021) were eligible for full-text review. A total of 288 articles met the inclusion criteria and were included. An overview of the characteristics of included studies can be found in Additional file 2.

### Pediatric patient engagement through the years

**Fig. 2:**
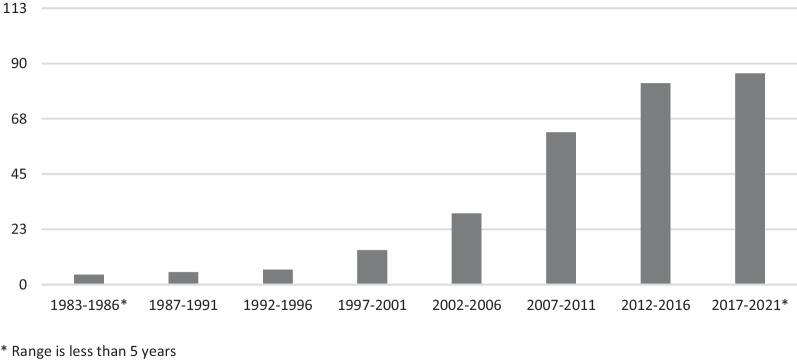
Overview of the included articles (number) per 5 years

The included articles are published between 1983 and February 2021, as shown in Fig. 2. Over the years there has been an increase in the number of studies that include pediatric patient engagement.

### Pediatric patient engagement per country

**Fig. 3:**
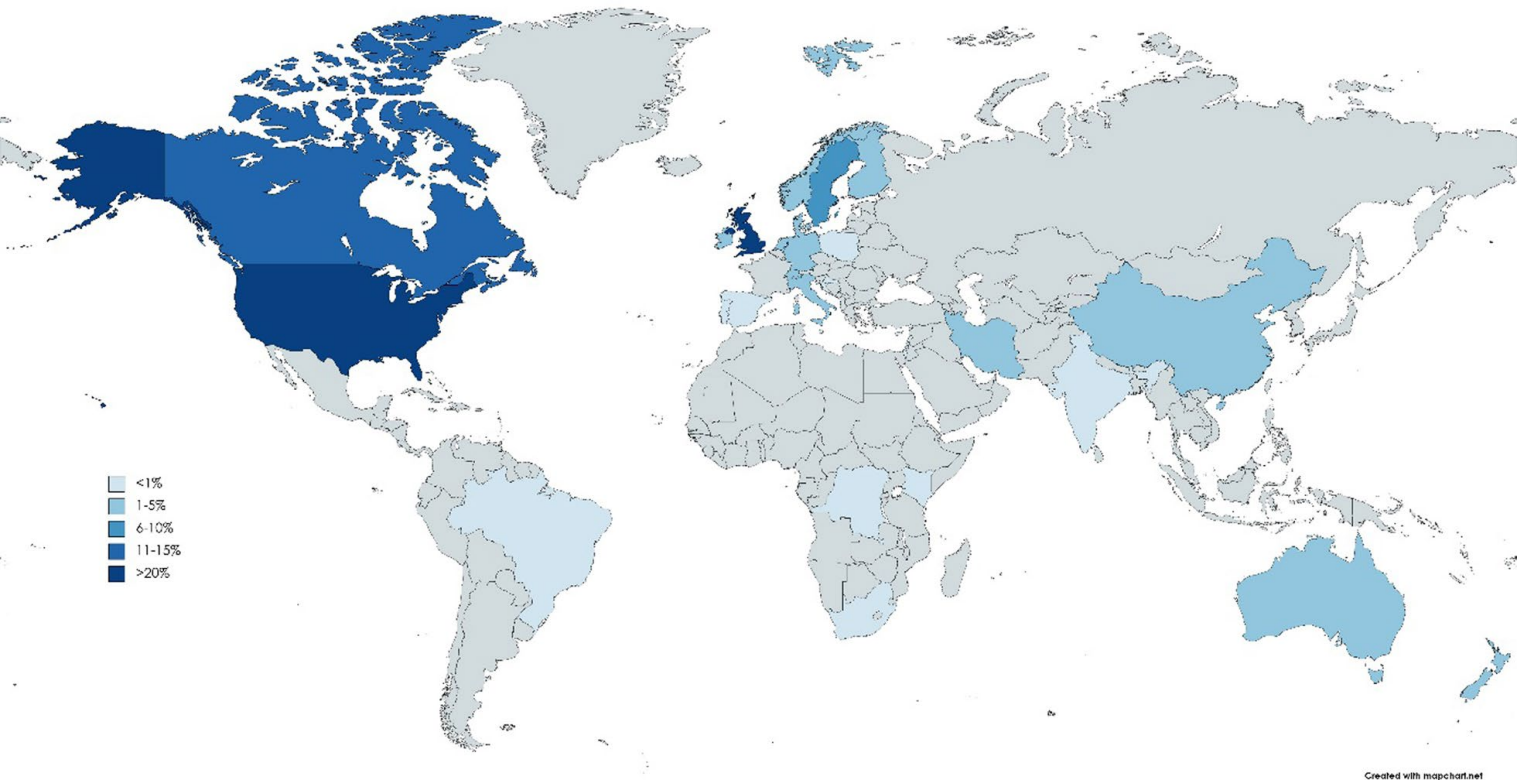
Overview of the articles (% of total) that include pediatric patient engagement per country

Figure 3 shows the number of studies in which pediatric patient engagement is included per country. Most studies involving pediatric patient engagement are performed in the United States of America, followed by the United Kingdom, and Canada.

### Pediatric patient engagement per disease group

The largest group of studied patients encompasses pediatric patients from different disease groups (26%) in their clinical care, research or development of intervention, and 10% of the studies concerned children being admitted to the hospital for various reasons. When looking at individual disease groups, pediatric oncology patients (22%) are most often engaged about their opinion, followed by pediatric patients undergoing surgery (7%), diabetes patients (5%), asthma patients (4%), transplant patients (4%), patients with Juvenile Idiopathic Arthritis (2%), and pediatric patients in palliative care (2%).

### Setting of patient engagement

Pediatric patients were asked for their opinion or experiences in different settings: in clinical care (81%), research (10%), and intervention development (9%). One study on adolescents’ beliefs about making treatment decisions and trial participation decisions following a cancer diagnosis was included in both the clinical care and research setting [21].

*Clinical care* The majority of the included articles were about pediatric patient engagement in clinical care. The aims of these studies were diverse. For example, children’s perspectives on the disclosure of medical errors were asked [22], children were asked about their experiences with postoperative pain and pain management [23], and adolescents’ preferred level of involvement in the decision-making process in cancer care was investigated [24]. The ultimate goal of engaging pediatric patients in these kind of studies was improving daily clinical care.

*Research* In the field of research, pediatric patients were mainly involved to gain more understanding into the reasons why pediatric patients do or do not participate in research, what factors influence their decision, and what adolescents’ preferences were regarding the organization of research participation [21, 25–28]. With this information, researchers aimed to improve recruitment strategies. In addition, a few studies evaluate the benefits and limitations of the use of a specific study design, for example a participatory research approach with chronically ill children as co-researchers [29], or asked pediatric patients with chronic conditions about their research priorities [30, 31].

*Intervention development* Pediatric patients were involved in the development and evaluation of various tools, such as a toolkit for advanced care planning [32], a therapeutic platform that provides health information to pediatric patients to prepare them for hospital procedures [33], a smartphone app developed to enhance medical adherence [34], and educational videos to motivate adolescents to become more actively involved during the outpatient visit [35].

### Used methods for pediatric patient engagement

**Fig. 4:**
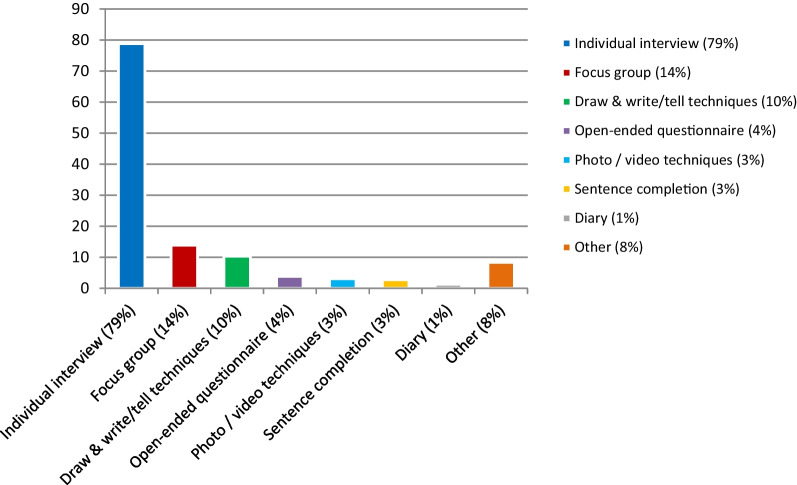
Overview of the methods used for pediatric patient engagement in the included articles

In the included articles different methods were used for pediatric patient engagement, as shown in Fig. 4. The most commonly used method to engage pediatric patients in clinical care, research and intervention development was an individual interview (227 studies), followed by focus groups (40 studies), and draw & write/tell techniques (30 studies). Other used methods were an open-ended questionnaire (11 studies), photo and video techniques (9 studies), sentence completion (8 studies), and keeping a diary (4 studies). Multiple methods were sometimes used in one study. Below is an overview of the different techniques used in the studies and examples of studies that used these methods to include pediatric patients in their projects.

### Individual interview

In individual interviews, the interviewer questions the pediatric patient about the experienced facts and perception of the topic of the research question [36]. In the included studies, pediatric patients were for example interviewed about their expectations regarding the quality of the nursing care [37] or about their experiences and wishes with regard to their first conversation about epilepsy with their clinician [38]. The interviews were conducted in different ways. Almost all studies used a semi-structured interview [38–40], but a few studies conducted an unstructured interview [41]. Furthermore, the majority of interviews were held face-to-face in the clinical setting [37–39] or at the patients’ home [40], and a few interviews were conducted by telephone [39].

### Focus group

A focus group is a group interview with several participants (the number of participants varies per study from 2 to 8 participants) [25, 36, 42, 43]. Focus groups were held about a wide variety of research questions, for example ‘What do adolescents with a rheumatic condition think about research involvement and how should adolescents involvement in research be organized? [25]’, ‘What are the perceptions and wishes of children with cancer regarding information exchange during their illness? [42]’ or ‘What do pediatric patients with life-limiting conditions think of the Implementing Pediatric Advance Care Planning Toolkit?’ [32]. Focus groups are often composed on shared characteristics, such as age or disease group, in order to obtain a homogeneous group [25, 44]. In most studies, focus groups are held with children from 11 years and older [25, 32, 43, 44]. An advantage of a focus group is that patients ask each other for explanations, resulting in more information in comparison to the sum of individual interviews. Disadvantages are that sometimes not every participant gets the chance to express their opinion due to the group composition and that experiences can be presented more polarized. An experienced discussion leader is necessary for a successful focus group [36].

### Draw & write/tell techniques

With the use of the draw & write/tell technique, pediatric patients are asked to draw a picture around the theme of the research question. The researcher uses the drawing as starting point for the conversation. An advantage of this technique is that the drawing increases the ability of children to talk about their experiences [45–47]. Most of the times, the draw & write/tell technique is used to ask for the experiences (e.g., experiences of children with regard to the treatment of recurrent cancer or to identify characteristics of a good nurse from the perspective of hospitalized children) of younger children (4–12 years) [45–47]. Draw & write/tell techniques are often used in combination with other quantitative or qualitative techniques [33, 47].

### Photo/video techniques

With photo/video techniques, pediatric patients are asked to choose/make photos or videos that represent their thoughts of feelings. For example, the things they did or did not like in the hospital [48, 49]. Subsequently, children are asked to provide an explanation to the pictures in an interview. An advantage of these techniques is that children are completely free to indicate what is important for them [48]. Examples of research questions for which photo/video techniques are used are ‘What are the experiences of adolescents living with type 1 diabetes, and what are their support needs during the transition from child- to adulthood’? [50] and ‘What are the experiences of children with the hospital care, and how could services be improved according to them?’ [48]. Photo/video techniques are used for a wide age group (from about 6 years) [48–50].

### Sentence completion

In this elicitation technique, patients are presented with half of a sentence and are asked to complete this. For example, the sentence started with *‘In my view, the best things about the hospital have been …*’. An advantage of the sentence completion technique is that it offers pediatric patients the opportunity to express their opinion in their own words, without being influenced by others [36, 51]. Sentence completion was used in studies that try to identify the experiences and wishes of pediatric patients with health care, with the ultimate goal to improve the quality of care [51, 52].

### Diary

Both unstructured and structured diaries can be used in study designs. With unstructured diaries, pediatric patients can write anything about a certain theme in their diary. While with the use of structured diaries, patients are asked to answer a number of questions on a daily basis. The included studies mainly used unstructured diaries in their research design [53]. Aims for which diaries are used are for example ‘Exploring the extent to which adolescents are involved in care planning’ and ‘Identify factors that affect pediatric patients while receiving pediatric palliative care’ [53, 54].

### Other

Other techniques that are used in the included studies to engage pediatric patients are, for example, participation in design meetings [55], advisory member of the research team, or other elicitation techniques like games, quizzes [56], and informal conversations [57].

### Patient engagement by age group

**Fig. 5:**
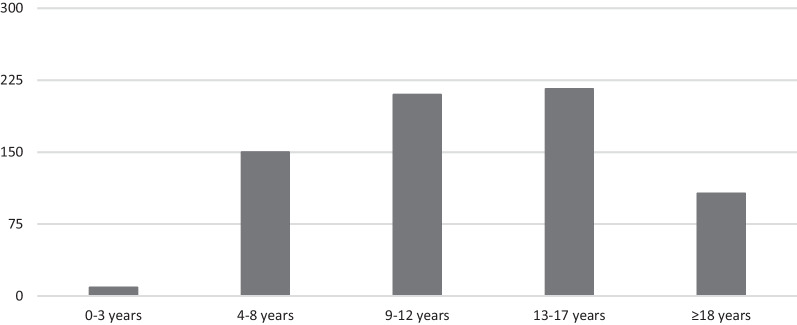
Overview of the number of articles that included pediatric patients in a specific age group

While some studies included young adults up to age 35 (some studies included both pediatric patients and young adults), analysis of engagement methods in this paper focuses on children up to age 18. In Fig. 5, an overview is provided of the number of studies that included pediatric patients in a specific age range. Pediatric patients in the age range 13–17 years were most often engaged in studies, followed by the age groups ranging from 9 to 12 years, and from 4 to 8 years. For 14 studies the age of the included pediatric patients was not clearly specified. The reason for this is in some cases that pediatric patient engagement has been conducted in a subset of the study population.

*2–3 years* The youngest age at which pediatric patients were involved in studies regarding clinical care, research or intervention development was 2 years. These young children were asked about their views of, for example, the hospital clown [58], their nurse or doctor [59], or their preferences for the used design/color in their hospital environment [60]. Except for one study (draw & write/tell technique) [59], interviewing was the used method for pediatric patient engagement in this age group. In most cases, parents were present to help their child or they were afterwards asked to reflect on the experiences of their child.

*4–8 years & 9–12 years* Pediatric patients in the age range 4–12 are regularly asked for their opinion in the health care setting. All described methods were used in this age group. The draw & write/tell technique is used more often in this age group compared to other age groups.

*13–17 years* Adolescent patients are most often included in pediatric patient engagement. Also in this age group, all described methods for patient engagement are used. However, focus groups were used more often in this age group compared to the younger age groups.

 ≥ *18 years* Most studies involved pediatric patients in their projects until the age of 18/19 years. Some studies involved a wider population and included both pediatric patients and young adults till the age of 35. This was the case, for example, in a study that aimed to establish a research agenda for patients with pediatric inflammatory bowel disease [30] or a study that investigated the views of adolescents and young adults (AYAs) with regard to their wishes and needs for a smart phone app that could be used to improve adherence to medication in the oncology setting [34].

## Discussion

This scoping review provided an overview of the existing literature about pediatric patient engagement in clinical care, research, and intervention development. The results showed an increase in the number of studies that report on pediatric patient engagement in the past decades, suggesting an increased interest in this topic. In the United States and Europe in particular, pediatric patients are more often involved in studies about clinical care, research, and intervention development compared to other countries and continents. A mix of patients from different disease groups were mostly asked for their opinion in the included studies, followed by oncology patients. Pediatric patients in the age range 9–17 years were most often engaged in a wide variety of projects compared to the other age groups. The individual interview is the most commonly used method to engage pediatric patients, followed by focus groups (for older children) and draw & write/tell techniques (for younger children). The majority of the included studies focused on the engagement of pediatric patients in clinical care with the aim to improve the quality of daily clinical care for patients.

The increased attention for pediatric patient engagement in the last decade is in line with the scoping review from Van Schelven et al. [3] about the involvement of adolescents (12–25 years) in research and implementation projects. Although our scoping review has a broader scope, included many studies, and focused on younger patients (4–18 years) in daily clinical care, the findings are comparable. Also in the study from Van Schelven et al. [3] the most important goal for patient engagement is improving the quality of care. In addition, the authors mentioned the lack of uniformity around the definition of patient engagement in the literature, which we underline. In the future, consensus needs to be reached about the definition of pediatric patient engagement and about the way clinicians and researchers should engage pediatric patients in their studies [3, 5, 9]. While conducting this study, it became evident that information on how pediatric patients were engaged was lacking. Therefore, we recommend, as a next step in the field, the development of a guideline to secure a uniform way to report on pediatric patient engagement in scientific papers. This guideline should include information on operationalization of patient engagement, goal, setting, age of patients, methods used, feasibility, and should be established in co-creation with all relevant stakeholders, definitely including patients and parents.

Regardless the external pressure/reinforcement (for example, pediatric patient engagement is increasingly mentioned as a requirement for grand applications by subsidy providers) for researchers to involve pediatric patients in their projects, only a few research projects include pediatric patients. This suggests that researchers need more tools and (financial) support to engage pediatric patients meaningfully. For example, we recently developed a patient engagement game for adolescents with a chronic condition, in cocreation with all stakeholders [61]. This game provides researchers and clinicians with a tool that can help them to engage pediatric patients meaningful in decision-making about clinical care, research and intervention development. In addition, we saw in some included studies that a small number of pediatric patients were involved in the project without having influences on the choices made, leading to tokenistic participation (a symbolic or perfunctory form of patient engagement, in which patients have no influence on decision-making [62]). Breaking through tokenism is difficult, as long as the added value and impact of pediatric patient engagement is not fully recognized, and challenges as funding, representativeness, changing power relations, and letting go of control over the project are not yet overcome [2, 3]. In addition, there are reasons and situations in which it may be particularly challenging or even inappropriate to engage children, because they may not have the capacity to understand some aspects of their care, and ultimately their parents can legally override their decisions about their own care.

Different methods were used to involve patients, with the individual interview being the most common method [9]. The methods used in pediatric patient engagement correspond with previous literature about patient engagement with both children and adult patients [3, 9]. Yet, there is no known best method to use for patient engagement. Which method is chosen depends on the project in which patients are involved, the age of the participants, and the availability of patients to participate [3, 9]. Future research should focus on increasing knowledge about the used methods and their suitability and impact for different research questions and target groups.

This scoping review provides a descriptive overview of the existing literature about pediatric patient engagement (4–18 years) in clinical care, research, and intervention development. This overview can inform clinicians or researchers, who are insecure about how to engage pediatric patients, about the different ways in which patient engagement can be shaped, and guide them to engage pediatric patients in their project. A strength of this study is the broad approach, making it possible to map the existing literature about pediatric patient engagement in a wide range of health care. However, due to its descriptive nature, the study also has a number of limitations. First, this study did not pay attention to the impact of pediatric patient engagement in the included studies. This might be an interesting area for future research as it could give us insight into the added value of patient engagement. Second, scoping reviews do not assess the quality of the included articles [16]. However, assessing the quality of studies could help us to better understand and interpret the results found. Third, due to geographical differences, pediatric care can be interpreted differently. Therefore, we did not include populations as dentistry and psychiatry. In addition, only articles published in English were included. Last, lack of uniformity about the definition of pediatric patient engagement and the influence of tokenism made it difficult to determine what exactly is done in the studies and whether patients actually influence the decision-making process. Therefore, it is possible that we missed studies in this review or that we incorrectly included studies.

In conclusion, this scoping review shows that there is an increasing interest in pediatric patient engagement. Pediatric patients are more often asked to express their views on questions in daily clinical care with the aim of improving the quality of care and tailoring care to patients’ needs. However, lack of uniformity about the definition of pediatric patient engagement and clear information and support for clinicians to engage patients in a meaningful way hinders engagement and can lead to tokenistic engagement. Guides, such as this overview, and sharing lessons learned can help clinicians to feel more confident about engaging pediatric patients in their daily practice.

## Supplementary Information

**Additional file 1**. Search strategy.

**Additional file 2**. Characteristics of the included studies.

## Data Availability

All data generated or analyzed during this study are included in this published article and its Additional files.

## Abbreviations

UNCRC: United Nations Convention on the Rights of the Child
VOS analysis: Visualization of similarities
