# Supplementary material for: Pediatric patient engagement in clinical care, research and intervention development: a scoping review
Source: J Patient Rep Outcomes. 2023 Mar 29;7:32. doi: 10.1186/s41687-023-00566-y (PMC10060502; doi:10.1186/s41687-023-00566-y)
Supplement: Supplementary file 2 — Additional file 2. Characteristics of the included studies. [file 41687_2023_566_MOESM2_ESM.docx]

| First author | **Year** | **Country** | ***N*** | **Disease group** | **Age (min)** | **Age (max)** | **Used method*** | **Setting** |
| --- | --- | --- | --- | --- | --- | --- | --- | --- |
| Abrines Jaume [1] | 2015 | United Kingdom | 36 | Multiple disease groups | 4 | 13 | 2 | Intervention development |
| Adams [2] | 2010 | Canada | 80 | Multiple disease groups | 5 | 18 | 1,4 | Clinical care |
| Akard [3] | 2013 | United States | 8 | Oncology | 7 | 12 | 1 | Intervention development |
| Alderfer [4] | 2017 | United States | 12 | Oncology | 12 | 25 | 1 | Clinical care |
| Alderson [5] | 2006 | United Kingdom | 24 | Diabetes | 3 | 12 | 1 | Clinical care |
| Alvarez [6] | 2020 | United States | 8 | Hospital admission | 7 | 15 | 1 | Clinical care |
| Ammerlaan [7] | 2015 | The Netherlands | 13 | Juvenile Idiopathic Arthritis | 16 | 25 | 1 | Clinical care |
| Anderson [8] | 2017 | United Kingdom | 6 | Transplantation | 15 | 18 | 1 | Clinical care |
| Anderzén-Carlsson [9] | 2012 | Sweden | 6 | Oncology | 14 | 16 | 1 | Clinical care |
| Angstrom-Brannstrom [10] | 2008 | Sweden | 7 | Multiple disease groups | 4 | 10 | 1,3 | Clinical care |
| Applebaum [11] | 2013 | United States | 20 | Multiple disease groups | 13 | 21 | 2 | Clinical care |
| Arruda-Colli [12] | 2015 | Brazil | 8 | Oncology | 5 | 12 | 3 | Clinical care |
| Arvidsson [13] | 2016 | Sweden | 5 | Oncology | 6 | 11 | 1,3,8 | Intervention development |
| Bagley [14] | 2007 | United States | 42 | Multiple disease groups | 5 | 15 | 1 | Research |
| Barned [15] | 2018 | Canada | 25 | Gastrointestinal diseases | 10 | 17 | 1 | Research |
| Baron [16] | 2011 | Australia | 26 | Oncology | 4;6 | 8;2 | 1 | Clinical care |
| Beck [17] | 2014 | Canada | 7 | Hematological disorders | 10 | 18 | 2 | Clinical care |
| Belsky [18] | 2020 | United States | 22 | Oncology | 15 | 30 | 1 | Intervention development |
| Belsky [19] | 2020 | United States | 20 | Oncology | 10 | 28 | 1 | Clinical care |
| Bemmels [20] | 2013 | United States | 10 | Surgery | 12 | 18 | 1 | Clinical care |
| Berkwitt [21] | 2015 | United States | 22 | Multiple disease groups | 7 | 18 | 1 | Clinical care |
| Bice [22] | 2019 | United States | 16 | Hospital admission | 4 | 8 | 1 | Intervention development |
| Black [23] | 1995 | Australia | 104 | Hospital admission | 12 | 18 | 1 | Clinical care |
| Board [24] | 2005 | United States | 21 | Hospital admission | 7 | 12 | 1 | Clinical care |
| Boisen [25] | 2015 | Denmark | NR | Oncology | NR | NR | 8 | Clinical care |
| Boyd [26] | 1998 | Canada | 6 | Multiple disease groups | 10 | 13 | 1,3,6 | Clinical care |
| Brady [27] | 2009 | United Kingdom | 22 | Hospital admission | 7 | 12 | 1,3 | Clinical care |
| Bray [28] | 2007 | United Kingdom | 7 | Surgery | 13 | 16 | 1,6 | Clinical care |
| Bray [29] | 2019 | United Kingdom | 32 | Multiple disease groups | 8 | 12 | 1 | Clinical care |
| Bray [30] | 2019 | United Kingdom | 106 | Multiple disease groups | 8 | 12 | 1,3 | Clinical care |
| Bray [31] | 2020 | United Kingdom | 80 | Multiple disease groups | 8 | 12 | 3 | Intervention development |
| Breitwieser [32] | 2014 | United States | 18 | Transplantation | 4 | 25 | 1,2,4 | Clinical care |
| Broome [33] | 2003 | United States | 23 | Multiple disease groups | 8 | 22 | 1 | Research |
| Brorsson [34] | 2017 | Sweden | 13 | Diabetes | 11;5 | 18 | 1 | Clinical care |
| Bsiri-Moghaddam [35] | 2011 | Iran | 12 | Surgery | 7 | 11 | 1 | Clinical care |
| Buckley [36] | 2010 | Ireland | 9 | Surgery | 6 | 9 | 1,3 | Clinical care |
| Burnfield [37] | 2019 | United States | 15 | Mobility problems | 3 | 11 | 8 | Clinical care |
| Byczkowski [38] | 2010 | United States | 170 | Multiple disease groups | 11 | 17 | 1 | Clinical care |
| Cahill [39] | 2019 | Australia | 200 | Multiple disease groups | 12 | 20 | 8 | Clinical care |
| Callery [40] | 2013 | United Kingdom | 13 | NR | NR | NR | 1 | Clinical care |
| Cameron [41] | 1983 | United States | 1 | Oncology | 11 | 11 | 8 | Intervention development |
| Carpenter [42] | 2014 | United States | 296 | Asthma | 8 | 16 | 1 | Clinical care |
| Castensoe-Seidenfaden [43] | 2016 | Denmark | 9 | Diabetes | 15 | 19 | 1,4 | Clinical care |
| Childerhose [44] | 2018 | United States | 7 | Obesity | 16 | 21 | 1 | Clinical care |
| Christofides [45] | 2016 | Canada | 19 | Cystic Fybrosis | 8 | 18 | 1 | Research |
| Chuong [46] | 2019 | Canada | 28 | Gastrointestinal diseases | 9 | 17 | 1 | Clinical care |
| Clark [47] | 2019 | United States | 40 | Hospital admission | 6 | 17 | 1,3 | Clinical care |
| Clarke [48] | 2020 | Ireland | 5 | Hospital admission | 10 | 11 | 2 | Intervention development |
| Clift [49] | 2007 | United Kingdom | 6 | Hospital admission | 11 | 15 | 1 | Clinical care |
| Coad [50] | 2008 | United Kingdom | 30 | Hospital admission | 3 | 18 | 1 | Clinical care |
| Corbo [51] | 1985 | United States | 14 | Patients on a ventilator | 12 | 16;9 | 1 | Clinical care |
| Corsano [52] | 2015 | Italy | 27 | Multiple disease groups | 6 | 15 | 1 | Clinical care |
| Coyne [53] | 2006 | United Kingdom | 11 | Multiple disease groups | 7 | 14 | 1 | Clinical care |
| Coyne [54] | 2006 | United Kingdom | 11 | Multiple disease groups | NR | NR | 1,5 | Clinical care |
| Coyne [55] | 2016 | Ireland | 20 | Oncology | 7 | 16 | 1 | Clinical care |
| Coyne [56] | 2014 | Ireland | 20 | Oncology | 7 | 16 | 1 | Clinical care |
| Coyne [57] | 2011 | Ireland | 55 | Multiple disease groups | 7 | 18 | 1,2 | Clinical care |
| Coyne [58] | 2012 | Ireland | 55 | Multiple disease groups | 7 | 18 | 1,5,8 | Clinical care |
| Crnkovic [59] | 2009 | Croatia | 190 | Multiple disease groups | 7 | 19 | 5 | Clinical care |
| Dagg [60] | 2020 | Canada | 7 | Surgery | 13 | 17 | 1 | Clinical care |
| Davies [61] | 2007 | United States | 4 | Palliative care | 6 | 17 | 1 | Clinical care |
| Davies [62] | 2005 | United States | 4 | Palliative care | 6 | 19 | 1 | Clinical care |
| Deatrick [63] | 1984 | United States | 24 | Surgery | NR | NR | 1 | Clinical care |
| Dedding [64] | 2015 | The Netherlands | 30 | Diabetes | 8 | 12 | 1,2 | Clinical care |
| Dekking [65] | 2015 | The Netherlands | 2 | Oncology | 13 | 18 | 1 | Research |
| Dell'Api [66] | 2007 | Canada | 5 | Chronic pain | 10 | 17 | 1 | Clinical care |
| Dellenmark-Blom [67] | 2016 | Sweden | 18 | Surgery | 8 | 17 | 2 | Intervention development |
| Di Ciommo [68] | 2012 | Italy | 20 | Metabolic diseases | 8 | 23 | 1 | Clinical care |
| Dixon-Woods [69] | 2002 | United Kingdom | 21 | Asthma | 8 | 16 | 1 | Clinical care |
| Dowler [70] | 2016 | United Kingdom | 25 | Hospital admission | NR | NR | 3 | Clinical care |
| Earle [71] | 2006 | Canada | 5 | Multiple disease groups | 4;5 | 17 | 1 | Clinical care |
| Ekra [72] | 2015 | Norway | 9 | Diabetes | 5 | 12 | 1,4 | Clinical care |
| Elliott [73] | 2019 | Canada | 2 | Juvenile Idiopathic Arthritis | NR | NR | 1,2 | Research |
| Erdem Atak [74] | 2019 | Cyprus | 31 | Multiple disease groups | 5 | 16 | 3 | Clinical care |
| Fahner [75] | 2020 | The Netherlands | 13 | Palliative care | 11 | 18 | 1,2 | Intervention development |
| Fallon [76] | 2008 | United Kingdom | 7 | Oncology | 14 | 23 | 2 | Intervention development |
| Farjou [77] | 2014 | Canada | 200 | Oncology | 12 | 20 | 7 | Clinical care |
| Ford [78] | 2011 | Australia | 10 | Surgery | 6 | 12 | 1 | Clinical care |
| Forsner [79] | 2005 | Sweden | 7 | Hospital admission | 7 | 10 | 1 | Clinical care |
| Foster [80] | 2019 | New Zealand | 26 | Hospital admission | 5 | 15 | 3 | Clinical care |
| Francis [81] | 1988 | United States | 7 | Multiple disease groups | 12 | 16 | 8 | Clinical care |
| Freeman [82] | 2000 | United States | 7 | Oncology | 5 | 15 | 1,2 | Clinical care |
| Gaab [83] | 2013 | New Zealand | 7 | Palliative care | 9 | 18 | 6 | Clinical care |
| Gagnon [84] | 2008 | Canada | 15 | Head / brain injury | 12 | 16 | 1 | Clinical care |
| Garth [85] | 2009 | Australia | 10 | Head / brain injury | 8 | 12 | 1 | Clinical care |
| Geehan [86] | 2003 | United Kingdom | 1 | Oncology | 17 | 17 | 8 | Clinical care |
| Gibson [87] | 2009 | New Zealand | 23 | Oncology | 12 | 22 | 2 | Clinical care |
| Gibson [88] | 2010 | United Kingdom | 38 | Oncology | 4 | 19 | 1,2,3,8 | Clinical care |
| Gillies [89] | 2001 | United Kingdom | 351 | Surgery | 12 | 18 | 1 | Clinical care |
| Gillis [90] | 1990 | Canada | 30 | Surgery | 6 | 12 | 1 | Clinical care |
| Gilljam [91] | 2016 | Sweden | 20 | Juvenile Idiopathic Arthritis | 8 | 17 | 1,2 | Clinical care |
| Gonzalez-Morkos [92] | 2011 | United States | 6 | Oncology | 15 | 18 | 8 | Clinical care |
| Grant [93] | 2019 | Canada | 25 | Gastrointestinal diseases | 11 | 35 | 2,8 | Research |
| Grinyer [94] | 2009 | United Kingdom | 28 | Oncology | 16 | 24 | 1 | Clinical care |
| Grover [95] | 2013 | India | 12 | Asthma | 7 | 12 | 1 | Clinical care |
| Haase [96] | 2020 | United States | 14 | Transplantation | 13 | 22 | 1 | Clinical care |
| Haase [97] | 1994 | United States | 7 | Oncology | 5 | 18 | 1 | Clinical care |
| Hall [98] | 2001 | United States | 34 | Multiple disease groups | 8 | 22 | 1 | Research |
| Hall [99] | 2012 | United Kingdom | 17 | Surgery | 8 | 17 | 1,3,8 | Clinical care |
| Han [100] | 2011 | China | 29 | Oncology | 7 | 14 | 1 | Clinical care |
| Hanghoj [101] | 2020 | Denmark | 12 | Oncology | 16 | 29 | 1 | Intervention development |
| Happ [102] | 2013 | United States | 11 | Cystic Fibrosis | 10 | 16 | 1 | Clinical care |
| Hart [103] | 2020 | United Kingdom | 18 | Oncology | 17 | 26 | 1 | Clinical care / research |
| Hein [104] | 2015 | The Netherlands | 35 | Multiple disease groups | 8 | 16 | 1 | Research |
| Heiney [105] | 1991 | United States | NR | Oncology | 13 | 22 | 2,8 | Clinical care |
| Hellier [106] | 1986 | United States | 20 | Radiology | 5 | 11 | 1 | Clinical care |
| Hentinen [107] | 1996 | Finland | 51 | Diabetes | 13 | 17 | 1 | Clinical care |
| Hoffman [108] | 2019 | United States | 21 | Ear, nose and throat disorders | 6 | 12 | 1 | Intervention development |
| Horstman [109] | 2002 | United Kingdom | 50 | Multiple disease groups | 6 | 10 | 1,3 | Clinical care |
| Hsiao [110] | 2007 | United States | 20 | Palliative care | 9 | 21 | 1 | Clinical care |
| Huby [111] | 2017 | United Kingdom | 26 | Kidney diseases | 5 | 17 | 1 | Intervention development |
| Huijer [112] | 2013 | Lebanon | 85 | Oncology | 7 | 18 | 1 | Clinical care |
| Hutton [113] | 2005 | Australia | 7 | Multiple disease groups | 13 | 18 | 1,3 | Clinical care |
| Ingersgaard [114] | 2017 | Denmark | 5 | Oncology | 14 | 19 | 1 | Research |
| Jamalimoghadam [115] | 2019 | Iran | 16 | Multiple disease groups | 12 | 18 | 1 | Clinical care |
| Jamalimoghadam [116] | 2019 | Iran | 13 | Multiple disease groups | 12 | 18 | 1 | Clinical care |
| Jedeloo [117] | 2010 | The Netherlands | 31 | Multiple disease groups | 12 | 19 | 1 | Clinical care |
| Jelbert [118] | 2010 | United Kingdom | 5 | Chronic Fatigue Syndrome | 13 | 18 | 1 | Clinical care |
| Jeschke [119] | 2020 | Germany | 101 | Epilepsy | 6 | 17 | 1 | Clinical care |
| Jiang [120] | 2020 | Australia | 14 | Juvenile Idiopathic Arthritis | 15 | 23 | 1 | Clinical care |
| Jibb [121] | 2017 | Canada | 16 | Oncology | 12 | 18 | 1,8 | Intervention development |
| Johnson [122] | 2008 | United States | 27 | Kidney diseases | NR | NR | 1 | Clinical care |
| Jolley [123] | 1992 | United Kingdom | 20 | Surgery | NR | NR | 7 | Clinical care |
| Jones [124] | 2001 | United States | 8 | Multiple disease groups | 13 | 17 | 2 | Research |
| Jordan [125] | 2019 | United States | 19 | Multiple disease groups | 13 | 19 | 1 | Clinical care |
| Jordan [126] | 2020 | Canada | 1 | Unknown | 17 | 17 | 8 | Clinical care |
| Kada [127] | 2019 | Norway | 22 | Radiology | 8 | 16 | 1 | Clinical care |
| Karande [128] | 2005 | India | 50 | Hospital admission | 5 | 12 | 1 | Clinical care |
| Karnieli-Miller [129] | 2009 | Israel | 13 | Gastrointestinal diseases | 9;6 | 16;6 | 1 | Clinical care |
| Kaziunas [130] | 2016 | United States | 17 | Transplantation | NR | NR | 1 | Clinical care |
| Kebbe [131] | 2019 | Canada | 19 | Obesity | 13 | 17 | 1 | Clinical care |
| Kebbe [132] | 2019 | Canada | 19 | Obesity | 13 | 17 | 1 | Clinical care |
| Kelly [133] | 2017 | United States | 29 | Oncology | 9 | 17 | 1,8 | Clinical care |
| Kelsey [134] | 2007 | United Kingdom | 10 | Multiple disease groups | 13 | 16 | 1 | Clinical care |
| Kiernan [135] | 2005 | Ireland | 240 | Multiple disease groups | 7 | 16 | 7 | Clinical care |
| King [136] | 2020 | Canada | 10 | Neuromuscular disorders | 8 | 18 | 1 | Clinical care |
| Klassen [137] | 2015 | Canada | 38 | Oncology | 15 | 26 | 1 | Intervention development |
| Kleye [138] | 2020 | Sweden | 13 | Multiple disease groups | 4 | 12 | 1,8 | Clinical care |
| Klosinski [139] | 2015 | Germany | 22 | Multiple disease groups | 12 | 17 | 1,2 | Intervention development |
| Klostermann [140] | 2005 | United States | 54 | Multiple disease groups | 11 | 19 | 2 | Clinical care |
| Koller [141] | 2016 | Canada | 26 | Multiple disease groups | 5 | 18 | 1 | Intervention development |
| Koller [142] | 2018 | Canada | 14 | Multiple disease groups | 9 | 17 | 2 | Clinical care |
| Koller [143] | 2010 | Canada | 21 | Multiple disease groups | 5 | 19 | 1 | Clinical care |
| Koller [144] | 2006 | Canada | 5 | Severe Acute Respiratory Syndrome | 6 | 18 | 1 | Clinical care |
| Kortesluoma [145] | 2008 | Finland | 44 | Hospital admission | 4 | 11 | 1 | Clinical care |
| Korus [146] | 2015 | Canada | 21 | Transplantation | 12 | 18 | 1 | Clinical care |
| Kulandaivelu [147] | 2018 | Canada | 19 | Sickle cell disease | 12 | 19 | 1 | Clinical care |
| Lam [148] | 2017 | China | 25 | Oncology | 9 | 18 | 1 | Clinical care |
| Lambert [149] | 2008 | Ireland | 49 | Hospital admission | 6 | 16 | 3,8 | Clinical care |
| Lambert [150] | 2013 | Ireland | 49 | Hospital admission | 6 | 16 | 3,8 | Clinical care |
| Laster [151] | 2009 | United States | 19 | Asthma | 8 | 17 | 2 | Clinical care |
| Leite [152] | 2019 | Brazil | 16 | Multiple disease groups | 7 | 12 | 1 | Clinical care |
| Lewis [153] | 2013 | United Kingdom | 30 | Epilepsy | 13 | 19 | 1,2 | Clinical care |
| Lindeke [154] | 2006 | United States | 120 | Hospital admission | 4 | 20 | 7 | Clinical care |
| Linder [155] | 2021 | United States | 19 | Oncology | 6 | 12 | 1 | Intervention development |
| Lindstrom Nilsson [156] | 2019 | Sweden | 50 | Multiple disease groups | 3 | 18 | 1 | Clinical care |
| Linge [157] | 2012 | Sweden | 9 | Multiple disease groups | 2 | 18 | 1 | Clinical care |
| Lipstein [158] | 2013 | United States | 15 | Multiple disease groups | 11 | 18 | 1 | Clinical care |
| Livesley [159] | 2013 | United Kingdom | 15 | Kidney diseases | 5 | 15 | 5 | Clinical care |
| Lock [160] | 2010 | United Kingdom | 12 | Ear, nose and throat disorders | 4 | 16 | 1 | Clinical care |
| Lööf [161] | 2019 | Sweden | 22 | Surgery | 4 | 15 | 1 | Clinical care |
| Lopez-Vargas [162] | 2018 | Australia | 3 | Multiple disease groups | 8 | 14 | 2 | Research |
| Loureiro [163] | 2020 | Portugal | 252 | Hospital admission | 7 | 11 | 5 | Clinical care |
| Luchtenberg [164] | 2015 | United Kingdom | 25 | Multiple disease groups | 10 | 23 | 1 | Research |
| Luchtenberg [165] | 2020 | United Kingdom / The Netherlands | 52 | Multiple disease groups | 9 | 18 | 1 | Research |
| Luseno [166] | 2017 | Kenya | 29 | Human Immunodeficiency Virus | 15 | 19 | 1 | Clinical care |
| Macartney [167] | 2014 | Canada | 12 | Oncology | 9 | 18 | 1 | Clinical care |
| Mansson [168] | 2013 | Sweden | 12 | Multiple disease groups | 5 | 10 | 1 | Clinical care |
| Mant [169] | 2019 | United Kingdom | 6 | Oncology | 8 | 12 | 1 | Clinical care |
| Marcinowicz [170] | 2016 | Poland | 22 | Multiple disease groups | 10 | 16 | 1 | Clinical care |
| Mari Ruberg Ekra [171] | 2012 | Norway | 9 | Diabetes | 7 | 12 | 1,4 | Clinical care |
| Martin-Kerry [172] | 2019 | United Kingdom | 21 | Multiple disease groups | 6 | 19 | 1,2 | Research |
| McCann [173] | 2014 | United Kingdom | 12 | Oncology | 17 | 25 | 1 | Clinical care |
| McDonald [174] | 2006 | United Kingdom | 63 | Hospital admission | 2 | 15 | 3 | Clinical care |
| Miller [175] | 1995 | United Kingdom | 7 | Multiple disease groups | 12 | 19 | 1 | Clinical care |
| Miller [176] | 2013 | United States | 20 | Oncology | 14 | 21 | 1 | Research |
| Mirlashari [177] | 2021 | Canada | 5 | Oncology | 13 | 15 | 1 | Clinical care |
| Mitchell [178] | 2014 | United States | 27 | Degenerative disorders | 13 | 22 | 1 | Clinical care |
| Morgan [179] | 2018 | United Kingdom | 3 | Oncology | 13 | 18 | 2 | Clinical care |
| Morgan [180] | 2009 | United Kingdom | 31 | Oncology | 13 | 24 | 7 | Clinical care |
| Morgan-Trimmer [181] | 2016 | United Kingdom | 7 | Diabetes | 7 | 16 | 1 | Clinical care |
| Morrow [182] | 2017 | Australia | 21 | Multiple disease groups | 7 | 17 | 1 | Intervention development |
| Moules [183] | 2009 | United Kingdom | 9 | Hospital admission | 12 | 16 | 1 | Clinical care |
| Nahata [184] | 2019 | United States | 14 | Oncology | 12 | 25 | 1 | Research |
| Needle [185] | 2020 | United States | 10 | Transplantation | 14 | 27 | 5 | Clinical care |
| Nguyen [186] | 2020 | United States / Canada | 32 | Transplantation | 12 | 24 | 2 | Clinical care |
| Nicholas [187] | 2007 | Canada | 9 | Multiple disease groups | 4 | 17 | 1 | Clinical care |
| Nicholas [188] | 2011 | Canada | 5 | Multiple disease groups | 4 | 17 | 1 | Clinical care |
| Nilsson [189] | 2010 | Sweden | 39 | Trauma | 5 | 10 | 1 | Clinical care |
| Noyes [190] | 2000 | United Kingdom | 18 | Patients on a ventilator | 6 | 18 | 1 | Clinical care |
| O'Callaghan [191] | 2011 | Australia | 26 | Oncology | 4;6 | 8;2 | 1 | Clinical care |
| Omondi [192] | 2013 | United States | 10 | Sickle cell disease | 12 | 16 | 2 | Research |
| Orr [193] | 2020 | Canada | 25 | Migraine | 10 | 18 | 1 | Clinical care |
| Owen [194] | 2013 | United Kingdom | 11 | Obesity | 5 | 18 | 1 | Clinical care |
| Padding [195] | 2018 | The Netherlands | 41 | Asthma | 5 | 6 | 1 | Clinical care |
| Paquette [196] | 2018 | United States | 20 | Hospital admission | 12 | 29 | 1 | Research |
| Parsons [197] | 2018 | United Kingdom | 63 | Juvenile Idiopathic Arthritis | 11 | 24 | 2 | Research |
| Patterson [198] | 2015 | United States | 29 | Sickle cell disease | 10 | 29 | 1 | Research |
| Pearce [199] | 2016 | United Kingdom | 21 | Oncology | 15 | 24 | 1 | Research |
| Pelander [200] | 2004 | Finland | 40 | Multiple disease groups | 4 | 11 | 1 | Clinical care |
| Pelander [201] | 2007 | Finland | 388 | Hospital admission | 7 | 11 | 3,7 | Clinical care |
| Pelander [202] | 2009 | Finland | 388 | Hospital admission | 7 | 11 | 1,3 | Intervention development |
| Pelander [203] | 2010 | Finland | 388 | Hospital admission | 7 | 11 | 5 | Clinical care |
| Pena [204] | 2014 | Spain | 30 | Multiple disease groups | 8 | 14 | 1 | Clinical care |
| Penza-Clyve [205] | 2004 | United States | 36 | Asthma | 9 | 15 | 2 | Clinical care |
| Perlman [206] | 1987 | Canada | 25 | Multiple disease groups | 4 | 7 | 1 | Clinical care |
| Perrott [207] | 2017 | United Kingdom | 249 | Surgery | NR | NR | 7 | Clinical care |
| Petronio-Coia [208] | 2020 | United States | 7 | Oncology | 8 | 12 | 1 | Clinical care |
| Pflugeisen [209] | 2019 | United States | 7 | Oncology | 16 | 34 | 8 | Clinical care |
| Picchietti [210] | 2010 | United States | 33 | Restless Legs Syndrome | 6 | 17 | 1 | Intervention development |
| Pichini [211] | 2016 | Canada | 11 | Genetic disorders | 12 | 18 | 1 | Clinical care |
| Polkki [212] | 1999 | Finland | 20 | Multiple disease groups | 7 | 11 | 1,8 | Clinical care |
| Pope [213] | 2018 | Australia | 15 | Acute pain | 4 | 8 | 1,3 | Clinical care |
| Pradel [214] | 2001 | United States | 32 | Asthma | 7 | 12 | 1,3 | Clinical care |
| Preti [215] | 2011 | Italy | 6 | Multiple disease groups | NR | NR | 1 | Clinical care |
| Przybylska [216] | 2019 | United Kingdom / Australia / United States | 9 | Hospital admission | 3 | 15 | 1 | Clinical care |
| Radovic [217] | 2018 | United States | 31 | Multiple disease groups | 13 | 18 | 1 | Clinical care |
| Ramsdell [218] | 2016 | United States | 10 | Trauma | 8 | 16 | 1 | Clinical care |
| Randall [219] | 2012 | United Kingdom | 21 | Multiple disease groups | 6 | 12 | 1,2,4 | Clinical care |
| Rasmussen [220] | 2017 | New Zealand | 6 | Hospital admission | NR | NR | 1 | Clinical care |
| Ray [221] | 2017 | United States | 2 | Multiple disease groups | 14 | 21 | 1 | Clinical care |
| Reverend Alister [222] | 2007 | United Kingdom | 5 | Multiple disease groups | 8 | 11 | 8 | Clinical care |
| Reynolds [223] | 2007 | United States | 32 | Diabetes | 12 | 17 | 1 | Research |
| Roper [224] | 2018 | United Kingdom | 16 | Asthma | 7 | 15 | 1 | Clinical care |
| Rosenberg [225] | 2016 | United States | 18 | Oncology | 14 | 25 | 1 | Clinical care |
| Ruhe [226] | 2016 | Switzerland | 17 | Oncology | 9 | 17 | 1 | Clinical care |
| Ruhe [227] | 2016 | Switzerland | 17 | Oncology | 9 | 17 | 1 | Clinical care |
| Ruland [228] | 2007 | Norway | 7 | Oncology | 8 | 12 | 1,2 | Intervention development |
| Runeson [229] | 2000 | Sweden | 26 | Multiple disease groups | 6 | 17 | 1 | Clinical care |
| Sartain [230] | 2000 | United Kingdom | 7 | Multiple disease groups | 8 | 14 | 1 | Clinical care |
| Sartain [231] | 2001 | United Kingdom | 11 | Hospital admission | 5 | 12 | 1,3 | Clinical care |
| Savedra [232] | 1988 | United States | 6 | Multiple disease groups | 13 | 16 | 1,4 | Clinical care |
| Schalkers [233] | 2015 | The Netherlands | 63 | Multiple disease groups | 6 | 18 | 1,4,5,8 | Clinical care |
| Schmidt [234] | 2007 | United States | 65 | Multiple disease groups | 5 | 18 | 1,7 | Clinical care |
| Schmidt [235] | 2012 | United States | 20 | Diabetes | 8 | 15 | 1 | Clinical care |
| Schwellnus [236] | 2020 | Canada | 10 | Head / brain injury | 6 | 19 | 1 | Clinical care |
| Sease [237] | 2021 | United States | 2 | Obesity | 6 | 18 | 1 | Clinical care |
| Sepponen [238] | 2003 | Finland | 31 | Asthma | 7 | 13 | 1 | Clinical care |
| Shaw [239] | 2006 | United Kingdom | 28 | Asthma | NR | NR | 1 | Clinical care |
| Sherratt [240] | 2018 | United Kingdom | 8 | Juvenile Idiopathic Arthritis | 8 | 16 | 1 | Research |
| Sjoberg [241] | 2015 | Sweden | 10 | Surgery | 8 | 11 | 1 | Clinical care |
| Skolin [242] | 2006 | Sweden | 22 | Oncology | 2 | 17 | 1 | Clinical care |
| Sleath [243] | 2016 | United States | 18 | Asthma | 11 | 17 | 2 | Intervention development |
| Smeland [244] | 2019 | Norway | 20 | Surgery | 8 | 16 | 1 | Clinical care |
| Smith [245] | 2005 | United Kingdom | 9 | Surgery | 7 | 11 | 3 | Clinical care |
| Smith [246] | 2019 | United States | 7 | Oncology | 7 | 17 | 2 | Clinical care |
| Squitieri [247] | 2013 | United States | 18 | Nerve damage | 10 | 17 | 1 | Clinical care |
| Stegenga [248] | 2013 | United States | 12 | Sickle cell disease | 6 | 14 | 1 | Clinical care |
| Stegenga [249] | 2018 | United States | 17 | Transplantation | 9 | 18 | 1 | Clinical care |
| Stegenga [250] | 2008 | United States | 3 | Oncology | 13 | 15 | 1 | Clinical care |
| Stegenga [251] | 2009 | United States | 10 | Oncology | 13 | 17 | 1 | Clinical care |
| Stevens [252] | 2006 | Canada | 14 | Oncology | 7 | 16 | 1 | Clinical care |
| Stevens [253] | 1998 | United States | 59 | Surgery | 12 | 17 | 1 | Clinical care |
| Stewart [254] | 2005 | United States | 6 | Oncology | 8 | 16 | 1 | Intervention development |
| Sutters [255] | 2007 | United States | 80 | Surgery | 6 | 15 | 1 | Clinical care |
| Taylor [256] | 2010 | United Kingdom | 21 | Multiple disease groups | 7 | 16 | 1 | Clinical care |
| Taylor [257] | 2016 | Australia | 26 | Head / brain injury | 6 | 15 | 7 | Intervention development |
| Tenniglo [258] | 2017 | The Netherlands | 11 | Oncology | 12 | 18 | 2 | Clinical care |
| Tercyak [259] | 1998 | United States | 12 | Diabetes | NR | NR | 1 | Clinical care |
| Tong [260] | 2013 | Australia | 13 | Juvenile Idiopathic Arthritis | 14 | 19 | 1,2 | Clinical care |
| Trace [261] | 2020 | United Kingdom | 5 | Kidney disease | 6 | 14 | 1 | Clinical care |
| Travlos [262] | 2016 | Australia | 11 | Neuromuscular disorders | 14 | 21 | 1 | Clinical care |
| Ullan [263] | 2012 | Spain | 126 | Multiple disease groups | 14 | 17 | 4,7 | Clinical care |
| Unguru [264] | 2010 | United States | 37 | Oncology | 7 | 19 | 7 | Research |
| Van Niekerk [265] | 2020 | South Africa | 15 | Burns | 9 | 15 | 2 | Clinical care |
| Van Staa [266] | 2010 | The Netherlands | 9 | Multiple disease groups | 15 | 17 | 1 | Research |
| Van Staa [267] | 2011 | The Netherlands | 31 | Multiple disease groups | 12 | 19 | 1 | Clinical care |
| Vaz [268] | 2010 | Congo | 8 | Human Immunodeficiency Virus | 8 | 17 | 1 | Clinical care |
| Vejzovic [269] | 2015 | Sweden | 17 | Gastrointestinal diseases | 10 | 17 | 1 | Clinical care |
| Viklund [270] | 2009 | Sweden | 31 | Diabetes | 12 | 17 | 1 | Clinical care |
| Visentin [271] | 2006 | Australia | 10 | Diabetes | 15 | 18 | 1 | Clinical care |
| Wangmo [272] | 2017 | Switzerland | 17 | Oncology | 9 | 17 | 1 | Clinical care |
| Weaver [273] | 2015 | United States | 40 | Oncology | 12 | 18 | 1 | Clinical care |
| Weaver [274] | 2020 | United States | 15 | Palliative care | NR | NR | 1 | Clinical care |
| Wennstrom [275] | 2008 | Sweden | 20 | Surgery | 6 | 9 | 1,3 | Clinical care |
| Wiener [276] | 2014 | United States | 24 | Transplantation | 5 | 17 | 1 | Intervention development |
| Wilkinson [277] | 2003 | United Kingdom | 14 | Oncology | 15 | 20 | 1,2 | Clinical care |
| Wilson [278] | 2010 | United States | 93 | Hospital admission | 5 | 9 | 3 | Clinical care |
| Wise [279] | 2002 | United States | 9 | Transplantation | 7 | 15 | 1 | Clinical care |
| Wollenhaupt [280] | 2012 | United States | 25 | Spina Bifida | 12 | 21 | 1 | Clinical care |
| Woltermann [281] | 2020 | Germany | 100 | Epilepsy | 6 | 18 | 1,3 | Clinical care |
| Woodgate [282] | 1995 | Canada | 11 | Acute pain | 2 | 6;5 | 1 | Clinical care |
| Woodgate [283] | 1998 | Canada | 23 | Multiple disease groups | 13 | 16 | 1 | Clinical care |
| Woodgate [284] | 2010 | Canada | 51 | Multiple disease groups | 6 | 19 | 1,2 | Research |
| Woodgate [285] | 2014 | Canada | 13 | Oncology | 8 | 17 | 1,3,6 | Clinical care |
| Woynarowska-Soldan [286] | 2015 | Poland | 24 | Multiple disease groups | 14 | 15 | 2 | Clinical care |
| Xie [287] | 2016 | China | 21 | Oncology | 7 | 12 | 1 | Clinical care |
| Zitzelsberger [288] | 2014 | Canada | 11 | Kidney diseases | 7 | 17 | 3,8 | Clinical care |

* 1 = individual interview, 2 = focus group, 3 = draw & write/tell techniques, 4 = photo/video techniques, 5 = complete sentences, 6 = diary, 7 = questionnaire with open questions, 8 = other

**References**

1. Abrines Jaume, N., Abbiss, M., Wray, J., Ashworth, J., Brown, K., & Cairns, J. (2015). CHILDSPLA: a collaboration between children and researchers to design and animate health states. *Child: Care, Health and Development, 41*(6), 1140-1151.

2. Adams, A., Theodore, D., Goldenberg, E., McLaren, C., & McKeever, P. (2010). Kids in the atrium: Comparing architectural intentions and children's experiences in a pediatric hospital lobby. *Social science & medicine, 70*(5), 658-667.

3. Akard, T. F., Gilmer, M. J., Friedman, D. L., Given, B., Hendricks-Ferguson, V. L., & Hinds, P. S. (2013). From qualitative work to intervention development in pediatric oncology palliative care research. *Journal of Pediatric Oncology Nursing, 30*(3), 153-160.

4. Alderfer, M. A., Lindell, R. B., Viadro, C. I., Zelley, K., Valdez, J., Mandrell, B., et al. (2017). Should genetic testing be offered for children? The perspectives of adolescents and emerging adults in families with Li-Fraumeni syndrome. *Journal of genetic counseling, 26*(5), 1106-1115.

5. Alderson, P. (2007). Competent children? Minors’ consent to health care treatment and research. *Social science & medicine, 65*(11), 2272-2283.

6. Alvarez, E. N., Pike, M. C., & Godwin, H. (2020). Children's and parents' views on hospital contact isolation: A qualitative study to highlight children's perspectives. *Clinical Child Psychology & Psychiatry, 25*(2), 401-418.

7. Ammerlaan, J. J., Scholtus, L. W., Drossaert, C. H., van Os-Medendorp, H., Prakken, B., Kruize, A. A., et al. (2015). Feasibility of a website and a hospital-based online portal for young adults with juvenile idiopathic arthritis: views and experiences of patients. *JMIR research protocols, 4*(3), e3952.

8. Anderson, S. M., Wray, J., Ralph, A., Spencer, H., Lunnon-Wood, T., & Gannon, K. (2017). Experiences of adolescent lung transplant recipients: A qualitative study. *Pediatric Transplantation, 21*(3).

9. Anderzén-Carlsson, A., Sörlie, V., & Kihlgren, A. (2012). Dealing with fear–from the perspective of adolescent girls with cancer. *European Journal of Oncology Nursing, 16*(3), 286-292.

10. Ångström-Brännström, C., Norberg, A., & Jansson, L. (2008). Narratives of children with chronic illness about being comforted. *Journal of pediatric nursing, 23*(4), 310-316.

11. Applebaum, M. A., Lawson, E. F., & von Scheven, E. (2013). Perception of transition readiness and preferences for use of technology in transition programs: teens’ ideas for the future. *International journal of adolescent medicine and health, 25*(2), 119-125.

12. Arruda-Colli, M., Perina, E., & Santos, M. (2015). Experiences of Brazilian children and family caregivers facing the recurrence of cancer. *European Journal of Oncology Nursing, 19*(5), 458-464.

13. Arvidsson, S., Gilljam, B.-M., Nygren, J., Ruland, C. M., Nordby-Bøe, T., & Svedberg, P. (2016). Redesign and validation of Sisom, an interactive assessment and communication tool for children with cancer. *JMIR mHealth and uHealth, 4*(2), e5715.

14. Bagley, S. J., Reynolds, W. W., & Nelson, R. M. (2007). Is a “wage-payment” model for research participation appropriate for children? *Pediatrics, 119*(1), 46-51.

15. Barned, C., Dobson, J., Stintzi, A., Mack, D., & O'Doherty, K. C. (2018). Children's perspectives on the benefits and burdens of research participation. *Ajob Empirical Bioethics, 9*(1), 19-28.

16. Baron, A., O'Callaghan, C., Barry, P., & Dun, B. MUSIC AND MUSIC THERAPY'S RELEVANCE FOR PAEDIATRIC CANCER PATIENTS AND THEIR FAMILIES: CONSTRUCTIVIST RESEARCH. In *Pediatric blood & cancer, 2011* (Vol. 57, pp. 855-855, Vol. 5): WILEY PERIODICALS, INC COMMERCE PLACE, 350 MAIN STREET, MALDEN, MA 02148-529 USA

17. Beck, C. E., Boydell, K. M., Stasiulis, E., Blanchette, V. S., Llewellyn-Thomas, H., Birken, C. S., et al. (2014). Shared decision making in the management of children with newly diagnosed immune thrombocytopenia. *Journal of pediatric hematology/oncology, 36*(7), 559-565.

18. Belsky, J. A., Holmes, C., Stanek, J., Yeager, N. D., & Audino, A. N. (2020). Evaluating Perspectives of a Smartphone Medication Application in the Adolescent and Young Adult Oncology Population: A Qualitative Study. *Journal of Adolescent & Young Adult Oncology, 22*, 22.

19. Belsky, J. A., Stanek, J., Skeens, M. A., Gerhardt, C. A., & Rose, M. J. (2021). Supportive care and osteopathic medicine in pediatric oncology: perspectives of current oncology clinicians, caregivers, and patients. *Supportive Care in Cancer, 29*(2), 1121-1128.

20. Bemmels, H., Biesecker, B., Schmidt, J. L., Krokosky, A., Guidotti, R., & Sutton, E. J. (2013). Psychological and social factors in undergoing reconstructive surgery among individuals with craniofacial conditions: an exploratory study. *The Cleft Palate-Craniofacial Journal, 50*(2), 158-167.

21. Berkwitt, A., & Grossman, M. (2015). A qualitative analysis of pediatric patient attitudes regarding family-centered rounds. *Hospital Pediatrics, 5*(7), 357-362.

22. Bice, A. A., Pond, R. S., & Lutz, B. J. (2019). The Pediatric Procedural Holistic Comfort Assessment: A Feasibility Study. *Journal of Pediatric Health Care, 33*(5), 509-519.

23. Black, W., Sawyer, M., & Fotheringham, M. (1995). Paediatric hospital services: are we satisfying our adolescent patients? *Journal of Quality in Clinical Practice, 15*(3), 161-167.

24. Board, R. (2005). School-age children's perceptions of their PICU hospitalization. *Pediatric nursing, 31*(3).

25. Boisen, K. A., Boisen, A., Thomsen, S. L., Matthiesen, S. M., Hjerming, M., & Hertz, P. G. (2015). Hacking the hospital environment: young adults designing youth-friendly hospital rooms together with young people with cancer experiences. *International journal of adolescent medicine and health, 29*(4).

26. Boyd, J. R., & Hunsberger, M. (1998). Chronically ill children coping with repeated hospitalizations: their perceptions and suggested interventions. *Journal of pediatric nursing, 13*(6), 330-342.

27. Brady, M. (2009). Hospitalized children’s views of the good nurse. *Nursing ethics, 16*(5), 543-560.

28. Bray, L. (2007). Experiences of young people admitted for planned surgery. *Nursing Children and Young People, 19*(5).

29. Bray, L., Appleton, V., & Sharpe, A. (2019). 'If I knew what was going to happen, it wouldn't worry me so much': Children's, parents' and health professionals' perspectives on information for children undergoing a procedure. *Journal of Child Health Care, 23*(4), 626-638.

30. Bray, L., Appleton, V., & Sharpe, A. (2019). The information needs of children having clinical procedures in hospital: Will it hurt? Will I feel scared? What can I do to stay calm? *Child: Care, Health & Development, 45*(5), 737-743.

31. Bray, L., Sharpe, A., Gichuru, P., Fortune, P. M., Blake, L., & Appleton, V. (2020). The Acceptability and Impact of the Xploro Digital Therapeutic Platform to Inform and Prepare Children for Planned Procedures in a Hospital: Before and After Evaluation Study. *Journal of Medical Internet Research, 22*(8), e17367.

32. Breitwieser, C. L., & Vaughn, L. M. (2014). “A Day in My life” Photography Project: The Silent Voice of Pediatric Bone Marrow Transplant Patients. *Journal of Pediatric Oncology Nursing, 31*(5), 284-292.

33. Broome, M. E., & Richards, D. J. (2003). The influence of relationships on children’s and adolescents’ participation in research. *Nursing research, 52*(3), 191-197.

34. Brorsson, A. L., Lindholm Olinder, A., Viklund, G., Granstrom, T., & Leksell, J. (2017). Adolescents' perceptions of participation in group education using the Guided Self-Determination-Young method: a qualitative study. *BMJ Open Diabetes Research & Care, 5*(1), e000432.

35. Bsiri-Moghaddam, K., Basiri-Moghaddam, M., Sadeghmoghaddam, L., & Ahmadi, F. (2011). The concept of hospitalization of children from the view point of parents and children. *Iranian Journal of Pediatrics, 21*(2), 201.

36. Buckley, A., & Savage, E. (2010). Preoperative information needs of children undergoing tonsillectomy. *Journal of clinical nursing, 19*(19‐20), 2879-2887.

37. Burnfield, J. M., Buster, T. W., Pfeifer, C. M., Irons, S. L., Cesar, G. M., & Nelson, C. A. (2019). Adapted motor-assisted elliptical for rehabilitation of children with physical disabilities. *Journal of Medical Devices, Transactions of the ASME, 13*(1).

38. Byczkowski, T. L., Kollar, L. M., & Britto, M. T. (2010). Family experiences with outpatient care: do adolescents and parents have the same perceptions? *Journal of Adolescent Health, 47*(1), 92-98.

39. Cahill, H., Wyn, J., & Borovica, T. (2019). Youth participation informing care in hospital settings. *Child & Youth Services, 40*(2), 140-157.

40. Callery, P., Kyle, R. G., Banks, M., Ewing, C., & Kirk, S. (2013). Enhancing parents' confidence to care in acute childhood illness: triangulation of findings from a mixed methods study of Community Children's Nursing. *Journal of Advanced Nursing, 69*(11), 2538-2548.

41. Cameron, C. O., & Wallace, N. (1983). Having a Bone Marrow Test: A Child's Perspective. *Children's Health Care, 12*(1), 41-42.

42. Carpenter, D. M., Stover, A., Slota, C., Ayala, G. X., Yeatts, K., Tudor, G., et al. (2014). An evaluation of physicians’ engagement of children with asthma in treatment-related discussions. *Journal of Child Health Care, 18*(3), 261-274.

43. Castensøe‐Seidenfaden, P., Teilmann, G., Kensing, F., Hommel, E., Olsen, B. S., & Husted, G. R. (2017). Isolated thoughts and feelings and unsolved concerns: adolescents’ and parents’ perspectives on living with type 1 diabetes–a qualitative study using visual storytelling. *Journal of clinical nursing, 26*(19-20), 3018-3030.

44. Childerhose, J. E., Eneli, I., & Steele, K. E. (2018). Adolescent bariatric surgery: a qualitative exploratory study of US patient perspectives. *Clinical Obesity, 8*(5), 345-354.

45. Christofides, E., Dobson, J. A., Solomon, M., Waters, V., & O’Doherty, K. C. (2016). Heuristic decision-making about research participation in children with cystic fibrosis. *Social science & medicine, 162*, 32-40.

46. Chuong, K. H., Haw, J., Stintzi, A., Mack, D. R., & O'Doherty, K. C. (2019). Dietary strategies and food practices of pediatric patients, and their parents, living with inflammatory bowel disease: a qualitative interview study. *International Journal of Qualitative Studies on Health and Well-being, 14*(1), 1648945.

47. Clark, M. E., Carleton, M. E., Cummings, B. M., & Noviski, N. (2019). Children's Drawings With Narratives in the Hospital Setting: Insights Into the Patient Experience. *Hospital Pediatrics, 9*(7), 495-500.

48. Clarke, S. (2020). Using Technology to Seek the Vulnerable and Marginalized Child's Voice in Hospital: Co-working with a 'Child Research Advisory Group' (CRAG). *Comprehensive Child & Adolescent Nursing*, 1-10.

49. Clift, L., Dampier, S., & Timmons, S. (2007). Adolescents' experiences of emergency admission to children's wards. *Journal of Child Health Care, 11*(3), 195-207.

50. Coad, J., & Coad, N. (2008). Children and young people's preference of thematic design and colour for their hospital environment. *Journal of Child Health Care, 12*(1), 33-48.

51. Corbo, B. H. (1985). Endotracheal intubation: adolescent ICU experiences. *Critical Care Quarterly*.

52. Corsano, P., Cigala, A., Majorano, M., Vignola, V., Nuzzo, M. J., Cardinale, E., et al. (2015). Speaking about emotional events in hospital: The role of health-care professionals in children emotional experiences. *Journal of Child Health Care, 19*(1), 84-92.

53. Coyne, I. (2006). Children's experiences of hospitalization. *Journal of Child Health Care, 10*(4), 326-336.

54. Coyne, I. (2006). Consultation with children in hospital: children, parents’ and nurses’ perspectives. *Journal of clinical nursing, 15*(1), 61-71.

55. Coyne, I., Amory, A., Gibson, F., & Kiernan, G. (2016). Information‐sharing between healthcare professionals, parents and children with cancer: more than a matter of information exchange. *European Journal of Cancer Care, 25*(1), 141-156.

56. Coyne, I., Amory, A., Kiernan, G., & Gibson, F. (2014). Children's participation in shared decision-making: children, adolescents, parents and healthcare professionals' perspectives and experiences. *European Journal of Oncology Nursing, 18*(3), 273-280.

57. Coyne, I., & Gallagher, P. (2011). Participation in communication and decision‐making: children and young people’s experiences in a hospital setting. *Journal of clinical nursing, 20*(15‐16), 2334-2343.

58. Coyne, I., & Kirwan, L. (2012). Ascertaining children’s wishes and feelings about hospital life. *Journal of Child Health Care, 16*(3), 293-304.

59. Crnković, M., Divčić, B., Rotim, Ž., & Čorić, J. (2009). Emotions and experiences of hospitalized school age patients. *Acta Clinica Croatica, 48*(2), 125-134.

60. Dagg, B., Forgeron, P., Macartney, G., & Chartrand, J. (2020). Adolescent Patients' Management of Postoperative Pain after Discharge: A Qualitative Study. *Pain Management Nursing, 21*(6), 565-571.

61. Davies, B., Collins, J., Steele, R., Cook, K., Distler, V., & Brenner, A. (2007). Parents’ and children's perspectives of a children's hospice bereavement program. *Journal of Palliative Care, 23*(1), 14-23.

62. Davies, B., Collins, J. B., Steele, R., Cook, K., Brenner, A., & Smith, S. (2005). Children's perspectives of a pediatric hospice program. *Journal of Palliative Care, 21*(4), 252-261.

63. Deatrick, J. A. (1984). It's their decision now: Perspectives of chronically disabled adolescents concerning surgery. *Issues in comprehensive pediatric nursing, 7*(1), 17-31.

64. Dedding, C., Reis, R., Wolf, B., & Hardon, A. (2015). Revealing the hidden agency of children in a clinical setting. *Health Expectations, 18*(6), 2121-2128.

65. Dekking, S. A., van der Graaf, R., Kars, M. C., Beishuizen, A., de Vries, M. C., & van Delden, J. J. (2015). Balancing research interests and patient interests: a qualitative study into the intertwinement of care and research in paediatric oncology. *Pediatric blood & cancer, 62*(5), 816-822.

66. Dell'Api, M., Rennick, J. E., & Rosmus, C. (2007). Childhood chronic pain and health care professional interactions: shaping the chronic pain experiences of children. *Journal of Child Health Care, 11*(4), 269-286.

67. Dellenmark‐Blom, M., Chaplin, J., Jönsson, L., Gatzinsky, V., Quitmann, J., & Abrahamsson, K. (2016). Coping strategies used by children and adolescents born with esophageal atresia–a focus group study obtaining the child and parent perspective. *Child: Care, Health and Development, 42*(5), 759-767.

68. Di Ciommo, V., Forcella, E., & Cotugno, G. (2012). Living with phenylketonuria from the point of view of children, adolescents, and young adults: a qualitative study. *Journal of Developmental & Behavioral Pediatrics, 33*(3), 229-235.

69. Dixon‐Woods, M., Anwar, Z., Young, B., & Brooke, A. (2002). Lay evaluation of services for childhood asthma. *Health & social care in the community, 10*(6), 503-511.

70. Dowler, L. (2016). Can improvised somatic dance reduce acute pain for young people in hospital? *Nursing Children and Young People, 28*(9).

71. Earle, R. J., Rennick, J. E., Carnevale, F. A., & Davis, G. M. (2006). ‘It's okay, it helps me to breathe’: The experience of home ventilation from a child's perspective. *Journal of Child Health Care, 10*(4), 270-282.

72. Ekra, E. M. R., Korsvold, T., & Gjengedal, E. (2015). Characteristics of being hospitalized as a child with a new diagnosis of type 1 diabetes: a phenomenological study of children’s past and present experiences. *BMC nursing, 14*(1), 1-10.

73. Elliott, L. K., Bami, H., Gelkopf, M. J., Yee, R. C., Feldman, B. M., & Goh, Y. I. (2019). Patient and caregiver engagement in research: factors that influence co-enrollment in research. *Pediatric Rheumatology Online Journal, 17*(1), 85.

74. Erdem Atak, I., Beyazit, U., Tascioglu, G., & Butun Ayhan, A. (2019). A study on the psychological status of hospitalized children and their perceptions of hospital and sickness through drawings. *Turkish Journal of Pediatric Disease, 13*(4), 283-291.

75. Fahner, J., Rietjens, J., van der Heide, A., Milota, M., van Delden, J., & Kars, M. (2021). Evaluation showed that stakeholders valued the support provided by the Implementing Pediatric Advance Care Planning Toolkit. *Acta Paediatrica, 110*(1), 237-246.

76. Fallon, S., Smith, J., Morgan, S., Stoner, M., & Austin, C. (2008). 'Pizza, patients and points of view': Involving young people in the design of a post registration module entitled the adolescent with cancer. *Nurse Education in Practice, 8*(2), 140-147.

77. Farjou, G., Sinha, R., Dix, D., Shahbaz, A., Klaassen, R. J., & Klassen, A. F. (2014). Understanding the healthcare experiences of teenaged cancer patients and survivors. *Child: Care, Health & Development, 40*(5), 723-730.

78. Ford, K. (2011). 'I didn't really like it, but it sounded exciting': admission to hospital for surgery from the perspectives of children. *Journal of Child Health Care, 15*(4), 250-260.

79. Forsner, M., Jansson, L., & Sorlie, V. (2005). The experience of being ill as narrated by hospitalized children aged 7-10 years with short-term illness. *Journal of Child Health Care, 9*(2), 153-165.

80. Foster, M., & Whitehead, L. (2019). Using drawings to understand the child's experience of child-centred care on admission to a paediatric high dependency unit. *Journal of Child Health Care, 23*(1), 102-117.

81. Francis, S., Myers-Gordon, K., & Pyper, C. (1988). Design of an adolescent activity room. *Children's Health Care, 16*(4), 268-273.

82. Freeman, K., O'Dell, C., & Meola, C. (2000). Issues in families of children with brain tumors. *Oncology Nursing Forum, 27*(5), 843-848.

83. Gaab, E. M., Owens, R. G., & MacLeod, R. D. (2013). The voices of young New Zealanders involved in pediatric palliative care. *Journal of Palliative Care, 29*(3), 186-192.

84. Gagnon, I., Swaine, B., Champagne, F., & Lefebvre, H. (2008). Perspectives of adolescents and their parents regarding service needs following a mild traumatic brain injury. *Brain Injury, 22*(2), 161-173.

85. Garth, B., Murphy, G. C., & Reddihough, D. S. (2009). Perceptions of participation: child patients with a disability in the doctor-parent-child partnership. *Patient Education & Counseling, 74*(1), 45-52.

86. Geehan, S. (2003). The benefits and drawbacks of treatment in a specialist Teenage Unit--a patient's perspective. *European Journal of Cancer, 39*(18), 2681-2683.

87. Gibson, C., & Nelson, K. (2009). Obtaining adolescents' views about inpatient facilities using conjoint analysis. *Paediatric Nursing, 21*(2), 34-37.

88. Gibson, F., Aldiss, S., Horstman, M., Kumpunen, S., & Richardson, A. (2010). Children and young people's experiences of cancer care: A qualitative research study using participatory methods. *International Journal of Nursing Studies, 47*(11), 1397-1407.

89. Gillies, M. L., Smith, L. N., & Parry-Jones, W. L. (2001). Postoperative pain: a comparison of adolescent inpatient and day patient experiences. *International Journal of Nursing Studies, 38*(3), 329-337.

90. Gillis, A. J. (1990). Hospital preparation: the children's story. *Children's Health Care, 19*(1), 19-27.

91. Gilljam, B. M., Arvidsson, S., Nygren, J. M., & Svedberg, P. (2016). Promoting participation in healthcare situations for children with JIA: a grounded theory study. *International Journal of Qualitative Studies on Health and Well-being, 11*, 30518.

92. Gonzalez-Morkos, B., Kuperberg, A., Zavala, O., Quinonez, B., & Booth, C. (2011). The teen impact experience: A web-casting pilot project for teens with cancer and blood diseases. *Psycho-Oncology, 1*, 42-43.

93. Grant, A., Crane, M., Laupacis, A., Griffiths, A., Burnett, D., Hood, A., et al. (2019). Engaging Patients and Caregivers in Research for Pediatric Inflammatory Bowel Disease: Top 10 Research Priorities. *Journal of Pediatric Gastroenterology & Nutrition, 69*(3), 317-323.

94. Grinyer, A. (2009). Contrasting parental perspectives with those of teenagers and young adults with cancer: Comparing the findings from two qualitative studies. *European Journal of Oncology Nursing, 13*(3), 200-206.

95. Grover, C., Goel, N., Chugh, K., Gaur, S. N., Armour, C., van Asperen, P. P., et al. (2013). Medication use in Indian children with asthma: the user's perspective. *Respirology, 18*(5), 807-813.

96. Haase, J. E., Robb, S. L., Burns, D. S., Stegenga, K., Cherven, B., Hendricks-Ferguson, V., et al. (2020). Adolescent/Young Adult Perspectives of a Therapeutic Music Video Intervention to Improve Resilience During Hematopoietic Stem Cell Transplant for Cancer. *Journal of Music Therapy, 57*(1), 3-33.

97. Haase, J. E., & Rostad, M. (1994). Experiences of completing cancer therapy: children's perspectives. *Oncology Nursing Forum, 21*(9), 1483-1492; discussion 1493-1484.

98. Hall, J. M., Stevens, P. E., & Pletsch, P. K. (2001). Team research using qualitative methods: Investigating children's involvement in clinical research. *Journal of Family Nursing, 7*(1), 7-31.

99. Hall, M., Gibson, B., James, A., & Rodd, H. D. (2012). Children's experiences of participation in the cleft lip and palate care pathway. *International Journal of Paediatric Dentistry, 22*(6), 442-450.

100. Han, J., Liu, J. E., Xiao, Q., Zheng, X. L., Ma, Y. H., & Ding, Y. M. (2011). The experiences and feelings of Chinese children living with leukemia: a qualitative study. *Cancer Nursing, 34*(2), 134-141.

101. Hanghoj, S., Pappot, H., Hjalgrim, L. L., Hjerming, M., Visler, C. L., & Boisen, K. A. (2020). Experiences of involvement processes during participation in cancer service user initiatives from an adolescent and young adult perspective. *International Journal of Adolescent Medicine & Health, 10*, 10.

102. Happ, M. B., Hoffman, L. A., DiVirgilio, D., Higgins, L. W., & Orenstein, D. M. (2013). Parent and child perceptions of a self-regulated, home-based exercise program for children with cystic fibrosis. *Nursing Research, 62*(5), 305-314.

103. Hart, R. I., Cameron, D. A., Cowie, F. J., Harden, J., Heaney, N. B., Rankin, D., et al. (2020). The challenges of making informed decisions about treatment and trial participation following a cancer diagnosis: a qualitative study involving adolescents and young adults with cancer and their caregivers. *BMC Health Services Research, 20*(1), 25.

104. Hein, I. M., Troost, P. W., de Vries, M. C., Knibbe, C. A., van Goudoever, J. B., & Lindauer, R. J. (2015). Why do children decide not to participate in clinical research: a quantitative and qualitative study. *Pediatric Research, 78*(1), 103-108.

105. Heiney, S. P., Wells, L. M., Coleman, B., & Swygert, E. (1991). "Lasting Impressions: adolescents with cancer share how to cope"--a videotape program. *Journal of Pediatric Oncology Nursing, 8*(1), 18-23.

106. Hellier, A., Ptak, H., & Cerreto, M. (1986). CATS inside my brain: children's understanding of the cerebral computed tomography scan procedure. *Children's Health Care, 14*(4), 211-217.

107. Hentinen, M., & Kyngas, H. (1996). Diabetic adolescents' compliance with health regimens and associated factors. *International Journal of Nursing Studies, 33*(3), 325-337.

108. Hoffman, M. F. (2019). Health-related quality of life instruments for deaf children with cochlear Implants: Development of child and parent-proxy measures. *Dissertation Abstracts International: Section B: The Sciences and Engineering, 80*(1), No Pagination Specified.

109. Horstman, M., & Bradding, A. (2002). Helping children speak up in the health service. *European Journal of Oncology Nursing, 6*(2), 75-84.

110. Hsiao, J. L., Evan, E. E., & Zeltzer, L. K. (2007). Parent and child perspectives on physician communication in pediatric palliative care. *Palliative & Supportive Care, 5*(4), 355-365.

111. Huby, K., Swallow, V., Smith, T., & Carolan, I. (2017). Children and young people's views on access to a web-based application to support personal management of long-term conditions: a qualitative study. *Child: Care, Health & Development, 43*(1), 126-132.

112. Huijer, H. A.-S., Sagherian, K., Tamim, H., Khoury, M. N., & Abboud, M. R. (2013). Quality of palliative care in children with cancer in Lebanon. *Lebanese Medical Journal, 103*(888), 1-9.

113. Hutton, A. (2005). Consumer perspectives in adolescent ward design. *Journal of Clinical Nursing, 14*(5), 537-545.

114. Ingersgaard, M. V., Tulstrup, M., Schmiegelow, K., & Larsen, H. B. (2018). A qualitative study of decision-making on Phase III randomized clinical trial participation in paediatric oncology: Adolescents' and parents' perspectives and preferences. *Journal of Advanced Nursing, 74*(1), 110-118.

115. Jamalimoghadam, N., Yektatalab, S., Momennasab, M., Ebadi, A., & Zare, N. (2019). How Do Hospitalized Adolescents Feel Safe? A Qualitative Study. *Journal of Nursing Research, 27*(2), e14.

116. Jamalimoghadam, N., Yektatalab, S., Momennasab, M., Ebadi, A., & Zare, N. (2019). Hospitalized adolescents' perception of dignity: A qualitative study. *Nursing Ethics, 26*(3), 728-737.

117. Jedeloo, S., van Staa, A., Latour, J. M., & van Exel, N. J. (2010). Preferences for health care and self-management among Dutch adolescents with chronic conditions: a Q-methodological investigation. *International Journal of Nursing Studies, 47*(5), 593-603.

118. Jelbert, R., Stedmon, J., & Stephens, A. (2010). A qualitative exploration of adolescents' experiences of chronic fatigue syndrome. *Clinical Child Psychology & Psychiatry, 15*(2), 267-283.

119. Jeschke, S., Woltermann, S., Neininger, M. P., Pauschek, J., Kiess, W., Bertsche, T., et al. (2020). Interviews with patients aged 6-17 years provide valuable insights for physicians who need to deliver an epilepsy diagnosis. *Acta Paediatrica, 18*, 18.

120. Jiang, I., Major, G., Singh-Grewal, D., Teng, C., Kelly, A., Niddrie, F., et al. (2021). Patient and parent perspectives on transition from paediatric to adult healthcare in rheumatic diseases: an interview study. *BMJ Open, 11*(1), e039670.

121. Jibb, L. A., Cafazzo, J. A., Nathan, P. C., Seto, E., Stevens, B. J., Nguyen, C., et al. (2017). Development of a mHealth real-time pain self-management app for adolescents with cancer: An iterative usability testing study. *Journal of Pediatric Oncology Nursing, 34*(4), 283-294.

122. Johnson, S., Sidelinger, D. E., Blanco, E., Palinkas, L. A., Macdonald, D., & Reznik, V. (2008). Ethnic differences and treatment trajectories in chronic kidney disease. *Journal of Health Care for the Poor & Underserved, 19*(1), 90-102.

123. Jolley, J. (1992). Parent and child views of an ENT ward. *Nursing Standard, 6*(34), 25-27.

124. Jones, F. C., & Broome, M. E. (2001). Focus groups with African American adolescents: enhancing recruitment and retention in intervention studies. *Journal of Pediatric Nursing, 16*(2), 88-96.

125. Jordan, A., Joseph-Williams, N., Edwards, A., Holland-Hart, D., & Wood, F. (2019). "I'd Like to Have More of a Say Because It's My Body": Adolescents' Perceptions Around Barriers and Facilitators to Shared Decision-Making. *Journal of Adolescent Health, 65*(5), 633-642.

126. Jordan, Z., Tremblay, C., Lipstein, E., Jordan, I., & Boland, L. (2020). Is sharing really caring? Viewpoints on shared decision-making in paediatrics. *Journal of Paediatrics & Child Health, 56*(5), 672-674.

127. Kada, S., Satinovic, M., Booth, L., & Miller, P. K. (2019). Managing discomfort and developing participation in non-emergency MRI: Children's coping strategies during their first procedure. *Radiography (London), 25*(1), 10-15.

128. Karande, S., Kelkar, A., & Kulkarni, M. (2005). Recollections of Indian children after discharge from an intensive care unit. *Pediatric Critical Care Medicine, 6*(3), 303-307.

129. Karnieli-Miller, O., & Eisikovits, Z. (2009). Physician as partner or salesman? Shared decision-making in real-time encounters. *Social Science & Medicine, 69*(1), 1-8.

130. Kaziunas, E., Hanauer, D. A., Ackerman, M. S., & Choi, S. W. (2016). Identifying unmet informational needs in the inpatient setting to increase patient and caregiver engagement in the context of pediatric hematopoietic stem cell transplantation. *Journal of the American Medical Informatics Association, 23*(1), 94-104.

131. Kebbe, M., Perez, A., Buchholz, A., McHugh, T. F., Scott, S. D., Richard, C., et al. (2019). End-user perspectives to inform policy and program decisions: a qualitative and quantitative content analysis of lifestyle treatment recommendations by adolescents with obesity. *BMC Pediatrics, 19*(1), 418.

132. Kebbe, M., Perez, A., Buchholz, A., Scott, S. D., McHugh, T.-L. F., Richard, C., et al. (2019). Adolescents' involvement in decision-making for pediatric weight management: A multi-centre, qualitative study on perspectives of adolescents and health care providers. *Patient Education and Counseling, 102*(6), 1194-1202.

133. Kelly, K. P., Mowbray, C., Pyke-Grimm, K., & Hinds, P. S. (2017). Identifying a conceptual shift in child and adolescent-reported treatment decision making: "Having a say, as I need at this time". *Pediatric Blood & Cancer, 64*(4), 04.

134. Kelsey, J., Abelson-Mitchell, N., & Skirton, H. (2007). Perceptions of young people about decision making in the acute healthcare environment. *Paediatric Nursing, 19*(6), 14-18.

135. Kiernan, G., Guerin, S., & MacLachlan, M. (2005). Children's voices: Qualitative data from the 'Barretstown studies'. *International Journal of Nursing Studies, 42*(7), 733-741.

136. King, G., Chiarello, L. A., Ideishi, R., D'Arrigo, R., Smart, E., Ziviani, J., et al. (2020). The Nature, Value, and Experience of Engagement in Pediatric Rehabilitation: Perspectives of Youth, Caregivers, and Service Providers. *Developmental neurorehabilitation, 23*(1), 18-30.

137. Klassen, A. F., Cano, S. J., Sinha, R., Shahbaz, A., Klaassen, R., & Dix, D. (2015). Is the Give Youth a Voice questionnaire an appropriate measure of teen-centred care in paediatric oncology: a Rasch measurement theory analysis. *Health Expectations, 18*(5), 1686-1697.

138. Kleye, I., Heden, L., Karlsson, K., Sundler, A. J., & Darcy, L. (2020). Children's individual voices are required for adequate management of fear and pain during hospital care and treatment. *Scandinavian Journal of Caring Sciences, 4*, 04.

139. Klosinski, M. G., & Farin, E. (2015). Communication preferences of chronically ill adolescents: development of an assessment instrument. *Psychological Assessment, 27*(3), 1053-1059.

140. Klostermann, B. K., Slap, G. B., Nebrig, D. M., Tivorsak, T. L., & Britto, M. T. (2005). Earning trust and losing it: adolescents' views on trusting physicians. *Journal of Family Practice, 54*(8), 679-687.

141. Koller, D. (2016). 'Kids need to talk too': inclusive practices for children's healthcare education and participation. *Journal of Clinical Nursing, 21*, 21.

142. Koller, D., & Espin, S. (2018). Views of children, parents, and health-care providers on pediatric disclosure of medical errors. *Journal of Child Health Care, 22*(4), 577-590.

143. Koller, D., Nicholas, D., Gearing, R., & Kalfa, O. (2010). Paediatric pandemic planning: children's perspectives and recommendations. *Health & Social Care in the Community, 18*(4), 369-377.

144. Koller, D. F., Nicholas, D. B., Goldie, R. S., Gearing, R., & Selkirk, E. K. (2006). Bowlby and Robertson revisited: the impact of isolation on hospitalized children during SARS. *Journal of Developmental & Behavioral Pediatrics, 27*(2), 134-140.

145. Kortesluoma, R. L., Nikkonen, M., & Serlo, W. (2008). "You just have to make the pain go away"--children's experiences of pain management. *Pain Management Nursing, 9*(4), 143-149, 149.e141-145.

146. Korus, M., Cruchley, E., Stinson, J. N., Gold, A., & Anthony, S. J. (2015). Usability testing of the Internet program: "Teens Taking Charge: Managing My Transplant Online". *Pediatric Transplantation, 19*(1), 107-117.

147. Kulandaivelu, Y., Lalloo, C., Ward, R., Zempsky, W. T., Kirby-Allen, M., Breakey, V. R., et al. (2018). Exploring the Needs of Adolescents With Sickle Cell Disease to Inform a Digital Self-Management and Transitional Care Program: Qualitative Study. *JMIR Pediatrics and Parenting, 1*(2), e11058.

148. Lam, K. K., Ho Cheung William, L., Ho, K. Y., Chung, O. K., & Chan, C. F. (2017). Factors contributing to the low physical activity level for Hong Kong Chinese children hospitalised with cancer: an exploratory study. *Journal of Clinical Nursing, 26*(1), 190-201.

149. Lambert, V., Glacken, M., & McCarron, M. (2008). 'Visible-ness': the nature of communication for children admitted to a specialist children's hospital in the Republic of Ireland. *Journal of Clinical Nursing, 17*(23), 3092-3102.

150. Lambert, V., Glacken, M., & McCarron, M. (2013). Meeting the information needs of children in hospital. *Journal of Child Health Care, 17*(4), 338-353.

151. Laster, N., Holsey, C. N., Shendell, D. G., McCarty, F. A., & Celano, M. (2009). Barriers to asthma management among urban families: caregiver and child perspectives. *Journal of Asthma, 46*(7), 731-739.

152. Leite, A., Alvarenga, W. A., Machado, J. R., Luchetta, L. F., Banca, R. O., Sparapani, V. C., et al. (2019). Children in outpatient follow-up: perspectives of care identified in interviews with puppet. *Revista Gaucha de Enfermagem, 40*, e20180103.

153. Lewis, S. A., & Noyes, J. (2013). Effective process or dangerous precipice: qualitative comparative embedded case study with young people with epilepsy and their parents during transition from children's to adult services. *BMC Pediatrics, 13*, 169.

154. Lindeke, L., Nakai, M., & Johnson, L. (2006). Capturing children's voices for quality improvement. *MCN, American Journal of Maternal Child Nursing, 31*(5), 290-295; quiz 296-297.

155. Linder, L. A., Newman, A. R., Stegenga, K., Chiu, Y. S., Wawrzynski, S. E., Kramer, H., et al. (2021). Feasibility and acceptability of a game-based symptom-reporting app for children with cancer: perspectives of children and parents. *Supportive Care in Cancer, 29*(1), 301-310.

156. Lindstrom Nilsson, M., Funkquist, E. L., Edner, A., & Engvall, G. (2020). Children report positive experiences of animal-assisted therapy in paediatric hospital care. *Acta Paediatrica, 109*(5), 1049-1056.

157. Linge, L. (2012). Magical attachment: Children in magical relations with hospital clowns. *International Journal of Qualitative Studies on Health and Well being, 7*.

158. Lipstein, E. A., Muething, K. A., Dodds, C. M., & Britto, M. T. (2013). "I'm the one taking it": adolescent participation in chronic disease treatment decisions. *Journal of Adolescent Health, 53*(2), 253-259.

159. Livesley, J., & Long, T. (2013). Children's experiences as hospital in-patients: voice, competence and work. Messages for nursing from a critical ethnographic study. *International Journal of Nursing Studies, 50*(10), 1292-1303.

160. Lock, C., Baker, R., & Brittain, K. (2010). 'I've just taken you to see the man with the CD on his head': the experience and management of recurrent sore throat in children. *Journal of Child Health Care, 14*(1), 95-110.

161. Loof, G., Andersson-Papadogiannakis, N., & Silen, C. (2019). Children's own perspectives demonstrate the need to improve paediatric perioperative care. *Nursing Open, 6*(4), 1363-1371.

162. Lopez-Vargas, P., Tong, A., Crowe, S., Alexander, S. I., Caldwell, P. H. Y., Campbell, D. E., et al. (2019). Research priorities for childhood chronic conditions: a workshop report. *Archives of Disease in Childhood, 104*(3), 237-245.

163. Loureiro, F. M., Antunes, A., Pelander, T., & Charepe, Z. B. (2020). The experience of school-aged children with hospitalisation. *Journal of Clinical Nursing, 25*, 25.

164. Luchtenberg, M., Maeckelberghe, E., Locock, L., Powell, L., & Verhagen, A. A. (2015). Young People's Experiences of Participation in Clinical Trials: Reasons for Taking Part. *American Journal of Bioethics, 15*(11), 3-13.

165. Luchtenberg, M. L., Maeckelberghe, E. L. M., Locock, L., & Verhagen, A. A. E. (2020). Understanding the child-doctor relationship in research participation: a qualitative study. *BMC Pediatrics, 20*(1), 353.

166. Luseno, W. K., Iritani, B., Zietz, S., Maman, S., Mbai, I., Otieno, F., et al. (2017). Experiences along the HIV care continuum: perspectives of Kenyan adolescents and caregivers. *African Journal of AIDS Research, 16*(3), 241-250.

167. Macartney, G., Stacey, D., Harrison, M. B., & VanDenKerkhof, E. (2014). Symptoms, coping, and quality of life in pediatric brain tumor survivors: a qualitative study. *Oncology Nursing Forum, 41*(4), 390-398.

168. Mansson, M. E., Elfving, R. N., Petersson, C., Wahl, J., & Tunell, S. (2013). Use of clowns to aid recovery in hospitalised children. *Nursing Children and Young People, 25*(10), 26-30.

169. Mant, J., Kirby, A., Cox, K. J., & Burke, A. (2019). Children's experiences of being diagnosed with cancer at the early stages of treatment; an interpretive phenomenological analysis. *Clinical Child Psychology & Psychiatry, 24*(1), 3-18.

170. Marcinowicz, L., Abramowicz, P., Zarzycka, D., Abramowicz, M., & Konstantynowicz, J. (2016). How hospitalized children and parents perceive nurses and hospital amenities: A qualitative descriptive study in Poland. *Journal of Child Health Care, 20*(1), 120-128.

171. Mari Ruberg Ekra, E., & Gjengedal, E. (2012). Being hospitalized with a newly diagnosed chronic illness-A phenomenological study of children's lifeworld in the hospital. *International Journal of Qualitative Studies on Health and Well-being, 7*(1), 18694.

172. Martin-Kerry, J. M., Knapp, P., Atkin, K., Bower, P., Watt, I., Stones, C., et al. (2019). Supporting children and young people when making decisions about joining clinical trials: qualitative study to inform multimedia website development. *BMJ Open, 9*(1), e023984.

173. McCann, L., Kearney, N., & Wengstrom, Y. (2014). "It's just going to a new hospital ... that's it." Or is it? An experiential perspective on moving from pediatric to adult cancer services. *Cancer Nursing, 37*(5), E23-E31.

174. McDonald, H., & Rushforth, H. (2006). Children's views of nursing and medical roles: implications for advanced nursing practice. *Paediatric Nursing, 18*(5), 32-36.

175. Miller, S. (1995). Adolescents' views of outpatient services. *Nursing Standard, 9*(17), 30-32.

176. Miller, V. A., Baker, J. N., Leek, A. C., Hizlan, S., Rheingold, S. R., Yamokoski, A. D., et al. (2013). Adolescent perspectives on phase I cancer research. *Pediatric Blood & Cancer, 60*(5), 873-878.

177. Mirlashari, J., Ebrahimpour, F., & Salisu, W. J. (2020). War on two fronts: Experience of children with cancer and their family during COVID-19 pandemic in Iran. *Journal of Pediatric Nursing, 57*, 25-31.

178. Mitchell, W. A. (2014). Making choices about medical interventions: the experience of disabled young people with degenerative conditions. *Health Expectations, 17*(2), 254-266.

179. Morgan, J. E., Phillips, B., Stewart, L. A., & Atkin, K. (2018). Quest for certainty regarding early discharge in paediatric low-risk febrile neutropenia: a multicentre qualitative focus group discussion study involving patients, parents and healthcare professionals in the UK. *BMJ Open, 8*(5), e020324.

180. Morgan, S. (2009). 'What colour is my cancer?' The experience of teenagers and young adults who are shown their cancer samples through a microscope. *European Journal of Oncology Nursing, 13*(3), 179-186 178p.

181. Morgan-Trimmer, S., Channon, S., Gregory, J. W., Townson, J., & Lowes, L. (2016). Family preferences for home or hospital care at diagnosis for children with diabetes in the DECIDE study. *Diabetic Medicine, 33*(1), 119-124.

182. Morrow, A. M., Burton, K. L., Watanabe, M. M., Cloyd, B. H., & Khut, G. P. (2018). Developing BrightHearts: A pediatric biofeedback-mediated relaxation app to manage procedural pain and anxiety. *Pain Practice, 18*(6), 698-708.

183. Moules, T. (2009). 'They wouldn't know how it feels...': characteristics of quality care from young people's perspectives: a participatory research project. *Journal of Child Health Care, 13*(4), 322-332.

184. Nahata, L., Morgan, T. L., Lipak, K. G., Clark, O. E., Yeager, N. D., O'Brien, S. H., et al. (2019). Conducting reproductive research during a new childhood cancer diagnosis: ethical considerations and impact on participants. *Journal of Assisted Reproduction & Genetics, 36*(9), 1787-1791.

185. Needle, J. S., Peden-McAlpine, C., Liaschenko, J., Koschmann, K., Sanders, N., Smith, A., et al. (2020). "Can you tell me why you made that choice?": A qualitative study of the influences on treatment decisions in advance care planning among adolescents and young adults undergoing bone marrow transplant. *Palliative Medicine, 34*(3), 281-290.

186. Nguyen, C., Dew, M., DeVito Dabbs, A., Irizarry, T., McNulty, M., & Foster, B. (2018). Promoting medication adherence from the perspectives of teen and young adult kidney recipients, parents and health care professionals. *American Journal of Transplantation, 18*, 706.

187. Nicholas, D. B., Darch, J., McNeill, T., Brister, L., O'Leary, K., Berlin, D., et al. (2007). Perceptions of online support for hospitalized children and adolescents. *Social Work in Health Care, 44*(3), 205-223.

188. Nicholas, D. B., Fellner, K. D., Koller, D., Fontana Chow, K., & Brister, L. (2011). Evaluation of videophone communication for families of hospitalized children. *Social Work in Health Care, 50*(3), 215-229.

189. Nilsson, S., Hallqvist, C., Sidenvall, B., & Enskar, K. (2011). Children's experiences of procedural pain management in conjunction with trauma wound dressings. *Journal of Advanced Nursing, 67*(7), 1449-1457.

190. Noyes, J. (2000). Enabling young 'ventilator-dependent' people to express their views and experiences of their care in hospital. *Journal of Advanced Nursing, 31*(5), 1206-1215.

191. O'Callaghan, C., Baron, A., Barry, P., & Dun, B. (2011). Music's relevance for pediatric cancer patients: a constructivist and mosaic research approach. *Supportive Care in Cancer, 19*(6), 779-788.

192. Omondi, N. A., Ferguson, S. E. S., Majhail, N. S., Denzen, E. M., Buchanan, G. R., Haight, A. E., et al. (2013). Barriers to hematopoietic cell transplantation clinical trial participation of African American and black youth with sickle cell disease and their parents. *Journal of Pediatric Hematology/Oncology, 35*(4), 289-298.

193. Orr, S. L., O'Sullivan, L., Zemek, R., Ward, N. M., & McMillan, H. J. (2020). Family perspectives on visiting the pediatric emergency department for migraine: A qualitative study. *Pediatric Emergency Care, 36*(6), e310-e317.

194. Owen, S. E., Sharp, D. J., Shield, J. P., & Turner, K. M. (2009). Childrens' and parents' views and experiences of attending a childhood obesity clinic: A qualitative study. *Primary Health Care Research and Development, 10*(3), 236-244.

195. Padding, A. M., Rutjes, N. W., Hashimoto, S., Vos, A., Staphorst, M. S., van Aalderen, W. M. C., et al. (2019). Young children experience little emotional burden during invasive procedures in asthma research. *European Journal of Pediatrics, 178*(2), 207-211.

196. Paquette, E. D., Derrington, S. F., Shukla, A., Sinha, N., Oswald, S., Sorce, L., et al. (2018). Biobanking in the Pediatric Critical Care Setting: Adolescent/Young Adult Perspectives. *Journal of Empirical Research on Human Research Ethics, 13*(4), 391-401.

197. Parsons, S., Thomson, W., Cresswell, K., Starling, B., & McDonagh, J. E. (2018). What do young people with rheumatic conditions in the UK think about research involvement? A qualitative study. *Pediatric Rheumatology, 16*(1), 35.

198. Patterson, C. A., Chavez, V., Mondestin, V., Deatrick, J., Li, Y., & Barakat, L. P. (2015). Clinical Trial Decision Making in Pediatric Sickle Cell Disease: A Qualitative Study of Perceived Benefits and Barriers to Participation. *Journal of Pediatric Hematology/Oncology, 37*(6), 415-422.

199. Pearce, S., Brownsdon, A., Fern, L., Gibson, F., Whelan, J., & Lavender, V. (2018). The perceptions of teenagers, young adults and professionals in the participation of bone cancer clinical trials. *European Journal of Cancer Care, 27*(6), e12476.

200. Pelander, T., & Leino-Kilpi, H. (2004). Quality in pediatric nursing care: children's expectations. *Issues in comprehensive pediatric nursing, 27*(3), 139-151.

201. Pelander, T., Leino-Kilpi, H., & Katajisto, J. (2007). Quality of pediatric nursing care in Finland: Children's perspective. *Journal of Nursing Care Quality, 22*(2), 185-194.

202. Pelander, T., Leino‐Kilpi, H., & Katajisto, J. (2009). The quality of paediatric nursing care: developing the Child Care Quality at Hospital instrument for children. *Journal of Advanced Nursing, 65*(2), 443-453.

203. Pelander, T., & Leino‐Kilpi, H. (2010). Children’s best and worst experiences during hospitalisation. *Scandinavian journal of caring sciences, 24*(4), 726-733.

204. Pena, A. L., & Rojas, J. G. (2014). Ethical aspects of children's perceptions of information-giving in care. *Nursing Ethics, 21*(2), 245-256.

205. Penza-Clyve, S. M., Mansell, C., & McQuaid, E. L. (2004). Why don't children take their asthma medications? A qualitative analysis of children's perspectives on adherence. *Journal of Asthma, 41*(2), 189-197.

206. Perlman, N., & Abramovitch, R. (1987). Visit to the pediatrician: children's concerns. *Journal of Pediatrics, 110*(6), 988-990.

207. Perrott, C., Lee, C. A., Griffiths, S., & Sury, M. R. J. (2018). Perioperative experiences of anesthesia reported by children and parents. *Paediatric Anaesthesia, 28*(2), 149-156.

208. Petronio-Coia, B. J., & Schwartz-Barcott, D. (2020). A description of approachable nurses: An exploratory study, the voice of the hospitalized child. *Journal of Pediatric Nursing, 54*, 18-23.

209. Pflugeisen, B. M., Patterson, P., Macpherson, C. F., Ray, B. C., Jacobsen, R. L., Hornyak, N., et al. (2019). Putting Adolescents and Young Adults in a Room Together: Launching an Adolescent and Young Adult Oncology Council. *Journal of Adolescent and Young Adult Oncology, 8*(5), 540-546.

210. Picchietti, D., Arbuckle, R., Abetz, L., Durmer, J., Ivanenko, A., Owens, J., et al. PEDIATRIC RESTLESS LEGS SYNDROME: QUALITATIVE ANALYSIS OF SYMPTOM DESCRIPTIONS AND DRAWINGS. In *Sleep, 2010* (Vol. 33, pp. A341-A342): AMER ACAD SLEEP MEDICINE ONE WESTBROOK CORPORATE CTR, STE 920, WESTCHESTER …

211. Pichini, A., Shuman, C., Sappleton, K., Kaufman, M., Chitayat, D., & Babul-Hirji, R. (2016). Experience with genetic counseling: the adolescent perspective. *Journal of Genetic Counseling, 25*(3), 583-595.

212. Polkki, T., Pietila, A. M., & Rissanen, L. (1999). Pain in children: qualitative research of Finnish school-aged children's experiences of pain in hospital. *International Journal of Nursing Practice, 5*(1), 21-28.

213. Pope, N., Tallon, M., Leslie, G., & Wilson, S. (2018). Ask me: Children's experiences of pain explored using the draw, write, and tell method. *Journal for Specialists in Pediatric Nursing: JSPN, 23*(3), e12218.

214. Pradel, F. G., Hartzema, A. G., & Bush, P. J. (2001). Asthma self-management: the perspective of children. *Patient Education & Counseling, 45*(3), 199-209.

215. Preti, C., & Welch, G. F. (2011). Music in a hospital: The impact of a live music program on pediatric patients and their caregivers. *Music and Medicine, 3*(4), 213-223.

216. Przybylska, M. A., Burke, N., Harris, C., Kazmierczyk, M., Kenton, E., Yu, O., et al. (2019). Delivery of the UN Convention on the Rights of the Child in an acute paediatric setting: an audit of information available and service gap analysis. *BMJ Paediatrics Open, 3*(1), e000445.

217. Radovic, A., McCarty, C. A., Katzman, K., & Richardson, L. P. (2018). Adolescents' Perspectives on Using Technology for Health: Qualitative Study. *JMIR Pediatrics and Parenting, 1*(1), e2.

218. Ramsdell, K. D., Morrison, M., Kassam-Adams, N., & Marsac, M. L. (2016). A Qualitative Analysis of Children's Emotional Reactions During Hospitalization Following Injury. *Journal of Trauma Nursing, 23*(4), 194-201.

219. Randall, D. (2012). Revisiting Mandell’s ‘least adult’role and engaging with children’s voices in research. *Nurse researcher, 19*(3).

220. Rasmussen, S., Water, T., & Dickinson, A. (2017). Children's perspectives in family-centred hospital care. *Contemporary Nurse, 53*(4), 445-455.

221. Ray, K. N., Ashcraft, L. E., Mehrotra, A., Miller, E., & Kahn, J. M. (2017). Family Perspectives on Telemedicine for Pediatric Subspecialty Care. *Telemedicine Journal & E-Health, 23*(10), 852-862.

222. Reverend Alister, B., & Gillies, M. (2007). Spiritual needs of children with complex healthcare needs in hospital. *Paediatric Nursing, 19*(9), 34-38.

223. Reynolds, W. W., & Nelson, R. M. (2007). Risk perception and decision processes underlying informed consent to research participation. *Social Science & Medicine, 65*(10), 2105-2115.

224. Roper, L., Sherratt, F. C., Young, B., McNamara, P., Dawson, A., Appleton, R., et al. (2018). Children's views on research without prior consent in emergency situations: a UK qualitative study. *BMJ Open, 8*(6), e022894.

225. Rosenberg, A. R., Bona, K., Wharton, C. M., Bradford, M., Shaffer, M. L., Wolfe, J., et al. (2016). Adolescent and Young Adult Patient Engagement and Participation in Survey-Based Research: A Report From the "Resilience in Adolescents and Young Adults With Cancer" Study. *Pediatric Blood & Cancer, 63*(4), 734-736.

226. Ruhe, K. M., Badarau, D. O., Brazzola, P., Hengartner, H., Elger, B. S., & Wangmo, T. (2016). Participation in pediatric oncology: views of child and adolescent patients. *Psycho-Oncology*, 1036-1042.

227. Ruhe, K. M., Wangmo, T., De Clercq, E., Badarau, D. O., Ansari, M., Kuhne, T., et al. (2016). Putting patient participation into practice in pediatrics-results from a qualitative study in pediatric oncology. *European Journal of Pediatrics, 175*(9), 1147-1155.

228. Ruland, C. M., Slaughter, L., Starren, J., Vatne, T. M., & Moe, E. Y. (2007). Children's contributions to designing a communication tool for children with cancer. *Studies in health technology and informatics, 129*(Pt 2), 977-982.

229. Runeson, I., , E., er, G., Hermeren, G., & Kristensson-Hallstrom, I. (2000). Children's consent to treatment: using a scale to assess degree of self-determination. *Pediatric Nursing, 26*(5), 455-458, 515.

230. Sartain, S. A., Clarke, C. L., & Heyman, R. (2000). Hearing the voices of children with chronic illness. *Journal of Advanced Nursing, 32*(4), 913-921.

231. Sartain, S. A., Maxwell, M. J., Todd, P. J., Haycox, A. R., & Bundred, P. E. (2001). Users' views on hospital and home care for acute illness in childhood. *Health & Social Care in the Community, 9*(2), 108-117.

232. Savedra, M. C., & Highley, B. L. (1988). Photography. Is it useful in learning how adolescents view hospitalization? *Journal of Adolescent Health Care, 9*(3), 219-224.

233. Schalkers, I., Dedding, C. W., & Bunders, J. F. (2015). '[I would like] a place to be alone, other than the toilet'--Children's perspectives on paediatric hospital care in the Netherlands. *Health Expectations, 18*(6), 2066-2078.

234. Schmidt, C., Bernaix, L., Koski, A., Weese, J., Chiappetta, M., , S., et al. (2007). Hospitalized children's perceptions of nurses and nurse behaviors. *MCN, American Journal of Maternal Child Nursing, 32*(6), 336-342; quiz 343-334.

235. Schmidt, C. A., Bernaix, L. W., Chiappetta, M., Carroll, E., , B., & , A. (2012). In-hospital survival skills training for type 1 diabetes: perceptions of children and parents. *MCN, American Journal of Maternal Child Nursing, 37*(2), 88-94.

236. Schwellnus, H., King, G., Baldwin, P., Keenan, S., & Hartman, L. R. (2020). A Solution-Focused Coaching Intervention with Children and Youth with Cerebral Palsy to Achieve Participation-Oriented Goals. *Physical & Occupational Therapy in Pediatrics, 40*(4), 423-440.

237. Sease, K., Griffin, S., Rolke, L., & Forrester, J. (2020). Feedback Following a Family-Focused Pediatric Weight Management Intervention: Experiences from the New Impact Program. *Pediatrics, 146*, 391-392.

238. Sepponen, K., Ahonen, R., & Vaskilampi, T. (2003). Children's perceptions of the use of asthma medicines - A qualitative interview study among Finnish children with asthma. *Journal of Social and Administrative Pharmacy, 20*(3), 92-102.

239. Shaw, A., Thompson, E. A., & Sharp, D. (2006). Complementary therapy use by patients and parents of children with asthma and the implications for NHS care: a qualitative study. *BMC Health Services Research, 6*, 76.

240. Sherratt, F. C., Roper, L., Stones, S. R., McErlane, F., Peak, M., Beresford, M. W., et al. (2018). Protective parents and permissive children: what qualitative interviews with parents and children can tell us about the feasibility of juvenile idiopathic arthritis trials. *Pediatric Rheumatology Online Journal, 16*(1), 76.

241. Sjoberg, C., Amhliden, H., Nygren, J. M., Arvidsson, S., & Svedberg, P. (2015). The perspective of children on factors influencing their participation in perioperative care. *Journal of Clinical Nursing, 24*(19), 2945-2953.

242. Skolin, I., Wahlin, Y. B., Broman, D. A., Koivisto Hursti, U. K., Vikstrom Larsson, M., & Hernell, O. (2006). Altered food intake and taste perception in children with cancer after start of chemotherapy: perspectives of children, parents and nurses. *Supportive Care in Cancer, 14*(4), 369-378.

243. Sleath, B., Carpenter, D. M., Lee, C., Loughlin, C. E., Etheridge, D., Rivera-Duchesne, L., et al. (2016). The development of an educational video to motivate teens with asthma to be more involved during medical visits and to improve medication adherence. *Journal of Asthma, 53*(7), 714-719.

244. Smeland, A. H., Rustoen, T., Naess, T., Nybro, L., Lundeberg, S., Reinertsen, H., et al. (2019). Children's views on postsurgical pain in recovery units in Norway: A qualitative study. *Journal of Clinical Nursing, 28*(11), 2157-2170.

245. Smith, L., & Callery, P. (2005). Children's accounts of their preoperative information needs. *Journal of Clinical Nursing, 14*(2), 230-238.

246. Smith, L. E., Maybach, A. M., Feldman, A., Darling, A., Akard, T. F., & Gilmer, M. J. (2019). Parent and child preferences and styles of communication about cancer diagnoses and treatment. *Journal of Pediatric Oncology Nursing, 36*(6), 390-401.

247. Squitieri, L., Larson, B. P., Chang, K. W., Yang, L. J., & Chung, K. C. (2013). Medical decision-making among adolescents with neonatal brachial plexus palsy and their families: a qualitative study. *Plastic & Reconstructive Surgery, 131*(6), 880e-887e.

248. Stegenga, K., & Burks, L. M. (2013). Using photovoice to explore the unique life perspectives of youth with sickle cell disease: A pilot study. *Journal of Pediatric Oncology Nursing, 30*(5), 269-274.

249. Stegenga, K., Pentz, R. D., Alderfer, M. A., Pelletier, W., Fairclough, D., & Hinds, P. S. (2019). Child and parent access to transplant information and involvement in treatment decision making. *Western Journal of Nursing Research, 41*(4), 576-591.

250. Stegenga, K., & Ward-Smith, P. (2008). The adolescent perspective on participation in treatment decision making: a pilot study. *Journal of Pediatric Oncology Nursing, 25*(2), 112-117.

251. Stegenga, K., & Ward-Smith, P. (2009). On receiving the diagnosis of cancer: The adolescent perspective. *Journal of Pediatric Oncology Nursing, 26*(2), 75-80.

252. Stevens, B., McKeever, P., Law, M. P., Booth, M., Greenberg, M., Daub, S., et al. (2006). Children receiving chemotherapy at home: perceptions of children and parents. *Journal of Pediatric Oncology Nursing, 23*(5), 276-285.

253. Stevens, M. S. (1988). Benefits of hospitalization: the adolescent's perspective. *Issues in Comprehensive Pediatric Nursing, 11*(4), 197-212.

254. Stewart, J. L., Lynn, M. R., & Mishel, M. H. (2005). Evaluating content validity for children's self-report instruments using children as content experts. *Nursing Research, 54*(6), 414-418.

255. Sutters, K. A., Savedra, M. C., Miaskowski, C., Holdridge-Zeuner, D., Waite, S., Paul, S. M., et al. (2007). Children's expectations of pain, perceptions of analgesic efficacy, and experiences with nonpharmacologic pain management strategies at home following tonsillectomy. *Journal for Specialists in Pediatric Nursing: JSPN, 12*(3), 139-148.

256. Taylor, S., Haase-Casanovas, S., Weaver, T., Kidd, J., & Garralda, E. M. (2010). Child involvement in the paediatric consultation: a qualitative study of children and carers' views. *Child: Care, Health & Development, 36*(5), 678-685.

257. Taylor, S., McLean, B., Parsons, R., Blair, E., Carey, L., Valentine, J., et al. (2016). Clinical acceptability of the SENSe assess kid: Children and youth perspectives. *Developmental Medicine and Child Neurology, 58*, 62-63.

258. Tenniglo, L. J. A., Loeffen, E. A. H., Kremer, L. C. M., Font-Gonzalez, A., Mulder, R. L., Postma, A., et al. (2017). Patients' and parents' views regarding supportive care in childhood cancer. *Supportive Care in Cancer*, 1-10.

259. Tercyak, K. P., Johnson, S. B., & Schatz, D. A. (1998). Patient and family reflections on the use of subcutaneous insulin to prevent diabetes: a retrospective evaluation from a pilot prevention trial. *Journal of Diabetes & its Complications, 12*(5), 279-286.

260. Tong, A., Jones, J., Speerin, R., Filocamo, K., Chaitow, J., & Singh-Grewal, D. (2013). Consumer perspectives on pediatric rheumatology care and service delivery: a qualitative study. *JCR: Journal of Clinical Rheumatology, 19*(5), 234-240.

261. Trace, S. L., Collinson, A., Searle, A. J., & Lithander, F. E. (2020). Using videoconsultations to deliver dietary advice to children with chronic kidney disease: a qualitative study of parent and child perspectives. *Journal of Human Nutrition & Dietetics, 33*(6), 881-889.

262. Travlos, V., Bulsara, C., Patman, S., & Downs, J. (2016). A fine balance and a shared learning journey: Exploring healthcare engagement through the experiences of youth with Neuromuscular Disorders. *Neurorehabilitation, 39*(4), 519-534.

263. Ullan, A. M., Belver, M. H., , F., ez, E., Serrano, I., Delgado, J., et al. (2012). Hospital designs for patients of different ages: Preferences of hospitalized adolescents, nonhospitalized adolescents, parents, and clinical staff. *Environment and Behavior, 44*(5), 668-694.

264. Unguru, Y., Sill, A. M., & Kamani, N. (2010). The experiences of children enrolled in pediatric oncology research: implications for assent. *Pediatrics, 125*(4), e876-e883.

265. Van Niekerk, A., Jacobs, R., Hornsby, N., Singh-Adriaanse, R., Sengoelge, M., & Laflamme, L. (2020). Enablers of psychosocial recovery in pediatric burns: perspectives from the children, parents and burn recovery support staff. *BMC Pediatrics, 20*(1), 289.

266. van Staa, A., Jedeloo, S., Latour, J. M., & Trappenburg, M. J. (2010). Exciting but exhausting: experiences with participatory research with chronically ill adolescents. *Health Expectations, 13*(1), 95-107.

267. van Staa, A., & On Your Own Feet Research, G. (2011). Unraveling triadic communication in hospital consultations with adolescents with chronic conditions: the added value of mixed methods research. *Patient Education & Counseling, 82*(3), 455-464.

268. Vaz, L. M., Eng, E., Maman, S., , T., u, T., & Behets, F. (2010). Telling children they have HIV: lessons learned from findings of a qualitative study in sub-Saharan Africa. *AIDS Patient Care & Stds, 24*(4), 247-256.

269. Vejzovic, V., Wennick, A., Idvall, E., & Bramhagen, A. C. (2015). A private affair: children's experiences prior to colonoscopy. *Journal of Clinical Nursing, 24*(7), 1038-1047.

270. Viklund, G., & Wikblad, K. (2009). Teenagers' perceptions of factors affecting decision-making competence in the management of type 1 diabetes. *Journal of Clinical Nursing, 18*(23), 3262-3270.

271. Visentin, K., Koch, T., & Kralik, D. (2006). Adolescents with Type 1 Diabetes: transition between diabetes services. *Journal of Clinical Nursing, 15*(6), 761-769.

272. Wangmo, T., De Clercq, E., Ruhe, K. M., Beck-Popovic, M., Rischewski, J., Angst, R., et al. (2017). Better to know than to imagine: Including children in their health care. *Ajob Empirical Bioethics, 8*(1), 11-20.

273. Weaver, M. S., Baker, J. N., Gattuso, J. S., Gibson, D. V., Sykes, A. D., & Hinds, P. S. (2015). Adolescents' preferences for treatment decisional involvement during their cancer. *Cancer, 121*(24), 4416-4424.

274. Weaver, M. S., Robinson, J. E., Shostrom, V. K., & Hinds, P. S. (2020). Telehealth Acceptability for Children, Family, and Adult Hospice Nurses When Integrating the Pediatric Palliative Inpatient Provider during Sequential Rural Home Hospice Visits. *Journal of Palliative Medicine, 23*(5), 641-649.

275. Wennstrom, B., Hallberg, L. R., & Bergh, I. (2008). Use of perioperative dialogues with children undergoing day surgery. *Journal of Advanced Nursing, 62*(1), 96-106.

276. Wiener, L., Baird, K., Crum, C., Powers, K., Carpenter, P., Baker, K. S., et al. (2014). Child and parent perspectives of the chronic graft-versus-host disease (cGVHD) symptom experience: a concept elicitation study. *Supportive Care in Cancer, 22*(2), 295-305.

277. Wilkinson, J. (2003). Young people with cancer--how should their care be organized? *European Journal of Cancer Care, 12*(1), 65-70.

278. Wilson, M. E., Megel, M. E., Enenbach, L., & Carlson, K. L. (2010). The voices of children: stories about hospitalization. *Journal of Pediatric Health Care, 24*(2), 95-102.

279. Wise, B. V. (2002). In their own words: the lived experience of pediatric liver transplantation. *Qualitative Health Research, 12*(1), 74-90.

280. Wollenhaupt, J., Rodgers, B., & Sawin, K. J. (2012). Family management of a chronic health condition: perspectives of adolescents. *Journal of Family Nursing, 18*(1), 65-90.

281. Woltermann, S., Jeschke, S., Herziger, B., Muller, R. M., Kiess, W., Bertsche, T., et al. (2020). Anticonvulsant long-term and rescue medication: The children's perspective. *European Journal of Paediatric Neurology, 28*, 180-185.

282. Woodgate, R., & Kristjanson, L. J. (1995). Young children's behavioural responses to acute pain: strategies for getting better. *Journal of Advanced Nursing, 22*(2), 243-249.

283. Woodgate, R. L. (1998). Health professionals caring for chronically ill adolescents: adolescents' perspectives. *Journal of the Society of Pediatric Nurses, 3*(2), 57-68.

284. Woodgate, R. L., & Edwards, M. (2010). Children in health research: a matter of trust. *Journal of Medical Ethics, 36*(4), 211-216.

285. Woodgate, R. L., West, C. H., & Tailor, K. (2014). Existential anxiety and growth: an exploration of computerized drawings and perspectives of children and adolescents with cancer. *Cancer Nursing, 37*(2), 146-159.

286. Woynarowska-Soldan, M., Tabak, I., Doroszewska, A., & Jablkowska-Gorecka, K. (2015). Teenagers' perception of being an active patient and putting the concept into practice. *Medycyna Wieku Rozwojowego, 19*(2), 202-211.

287. Xie, A., Shan, Y., Niu, M. E., Chen, Y., & Wang, X. (2016). Experience and nursing needs of school-age children undergoing lumbar puncture during the treatment of acute lymphoblastic leukaemia: a descriptive and qualitative study. *Journal of Clinical Nursing, 1*, 01.

288. Zitzelsberger, H., McKeever, P., Peter, E., Chambon, A., Morgan, K. P., & Spalding, K. (2014). Doing 'technological time' in a pediatric hemodialysis unit: an ethnography of children. *Health & Place, 27*, 112-119.
